# Comprehensive prognostic and immunological analysis of Ubiquitin Specific Peptidase 28 in pan-cancers and identification of its role in hepatocellular carcinoma cell lines

**DOI:** 10.18632/aging.204869

**Published:** 2023-07-13

**Authors:** Wuhan Zhou, Jiafei Chen, Jingui Wang

**Affiliations:** 1 Department of Hepatobiliary Surgery, The First Hospital of Putian City, Putian 351100, Fujian, China; 2 Department of Clinical Medicine, Fujian Medical University, Fuzhou 350108, Fujian, China

**Keywords:** immune checkpoint, prognosis, immunologic infiltration, molecular biology experiments, pan-cancer

## Abstract

Background: Ubiquitin Specific Peptidase 28 (USP28), as a member of the DUBs family, has been reported to regulate the occurrence and development of some tumors, but its oncogenic role in tumor immunity is still unknown.

Methods: The comprehensive view of USP28 expression in tumor and normal samples was obtained from public databases, including The Cancer Genome Atlas (TCGA), Genotype-Tissue Expression (GTEx), and Cancer Cell Line Encyclopedia (CCLE). We analyzed the genomic alterations of USP28 in various cancers using the cBioPortal dataset. Besides, gene set enrichment analysis was used to analyze the associated cancer hallmarks with USP28 expression, and TIMER2.0 was taken to investigate the immune cell infiltrations related to the USP28 level.

Results: USP28 is highly expressed in most tumors and has prognostic value across various cancer types. Moreover, a significant correlation exists between USP28 and immune regulators, clinical staging, checkpoint inhibitor response, MSI, TMB, CNV, MMR defects, and DNA methylation. Additionally, USP28 expression is strongly associated with the infiltration levels of neutrophils and NK cells in most tumor types. One of the most significant findings of our study was that USP28 could serve as a significant predictor of anti-CTLA4 therapy response in melanoma patients. Additionally, our molecular biology experiments validated that the knockdown of USP28 substantially reduced the proliferative and invasive abilities of the HCC cell lines.

Conclusions: Our study suggests that USP28 could potentially serve as a biomarker for cancer immunologic infiltration and poor prognosis, with potential applications in developing novel cancer treatment strategies.

## INTRODUCTION

The incidence of malignant tumors has indeed been increasing at an alarming rate over the past few decades, which has become a leading cause of human death and a major worldwide public health burden [1]. Immune checkpoint blockade has significantly contributed to cancer patients’ immunotherapy. However, drug resistance to immunotherapy is still a major challenge that needs to be addressed urgently. Pan-cancer analyses have been carried out smoothly using continuously accumulating multi-omics data across cancer types [2]. Unlike conventional single-type tumor research, pan-cancer analysis can show the similarity and heterogeneity of various tumors and provide a broad overview of genetic variation, tumor microenvironment, and immunotherapy [3]. As a result, research and discovery of new immunotherapy biomarkers or immunoregulatory genes will have important clinical significance for the efficacy of immunotherapy in cancer patients.

Ubiquitin-specific peptidase 28 (USP28) is a deubiquitinase (DUB) enzyme belonging to the USP family, which was discovered to be closely related to cell-cycle progression, DNA repair, apoptosis, proliferation, and tumorigenesis [4–6], which suggested USP28 might be a promising target for cancer therapy. The human USP28 gene is located on chromosome 11q23, ending in a protein with 1077 amino acids [7]. The expression level of USP28 was relevant to the poor prognosis of some cancers, including colon cancer [8], bladder cancer [9], and non-small-cell lung cancer (NSCLC) [10].

USP28 plays an indispensable role in tumor progression by regulating multiple signaling pathways. For example, USP28 facilitates pancreatic cancer progression through activation of the Wnt/β-catenin pathway via stabilizing FOXM1 [11] USP28 promotes colorectal cancer progression by increasing FOXC1 stability [12]. Similarly, USP28 also controls intestinal homeostasis and promotes colorectal cancer [13]. In addition, USP28 also plays an important role in the progression of squamous cell lung cancer [14]. Also, USP28 is closely related to cell proliferation and metastasis in breast cancer [15]. Given that USP28 plays an indispensable role in different tumor progression, it is particularly important to systematically and holistically explore the role of USP28 in pan-cancer. Therefore, targeting deubiquitinase USP28 for cancer therapy is very important [4]. Currently, there is no all-encompassing investigation that thoroughly explains the impact of USP28 on tumor immune infiltration and response to immunotherapy across multiple types of cancer. In this study, we used public databases to explore the expression, mutation, and prognosis profiles of USP28 in various cancers. Our data imply that the USP28 expression level was increased in most cancer tumors and confirmed that it is highly expressed in HCC clinical samples. Furthermore, the relationship between USP28 and genomic alterations, prognosis, Gene Set Enrichment Analysis (GSEA), and immune cell infiltration analysis suggests that USP28 has the potential to serve as a valuable biomarker for immunotherapy. In addition, we performed molecular biology experiments in HCC cell lines to further validate the oncogenic function of USP28. In summary, USP28 represents a promising and potential therapeutic target for cancer treatment, indicating immune infiltration and unfavorable prognosis in cancer patients.

## MATERIALS AND METHODS

### Data collection

We analyzed the expression patterns of USP28 comprehensively by utilizing public datasets such as the Cancer Genome Atlas (TCGA) [16], the Genotype-Tissue Expression (GTEx) [17], Cancer Cell Line Encyclopedia (CCLE) [18], and Clinical proteomic tumor analysis Consortium (CPTAC) [19] databases. Log2 transformation was performed to normalize the expression data. In addition, we also analyzed the phosphorylation (with phosphorylation at the Y1117, S1115, S896, S113, S495, S1115, and S896 sites) of USP28 (NP_001333181.1) between primary tumor and normal tissues, respectively based on the CPTAC dataset via the UALCAN portal (http://ualcan.path.uab.edu/analysis-prot.html) [20]. Moreover, the promoter methylation level of USP28 in pan-cancer was analyzed by the TCGA dataset via the UALCAN portal [21]. Supplementary Table 1 provides the abbreviations of the cancers included in this study.

### Single-cell analysis of USP28

We utilized the Tumor Immune Single-cell Hub (TISCH) web tool to perform our single-cell analysis. The heatmap and scatter plots were used to quantify and visualize the expression levels of USP28 in various cell types. Details regarding data collection, processing, and cell annotation procedures can be found in the documentation section of the TISCH website (http://tisch.comp-genomics.org/documentation/) [22].

### Genetic alteration analysis of USP28

cBioPortal for Cancer Genomics (http://cbioportal.org) [23] was used to observe the genomic alteration frequency, mutation type, copy number alteration, and mutation count in mutation types of USP28 in all TCGA tumors. The mutated site information of USP28 was obtained in the schematic diagram of the protein structure. The Three-dimensional of the mutated site was also displayed in the “Mutation” module.

### Relationship between USP28 expression and clinical stage, MMR, and methyltransferases

We used the “Pathological Stage Plot” module of GEPIA2 (http://gepia2.cancer-pku.cn/#analysis) to analyze the expression of USP28 across different stages (stage I, stage II, stage III, and stage IV) in all TCGA tumors [24]. The box plots were created using log2 [TPM (Transcripts per million) +1] transformed expression data. Furthermore, utilizing the TCGA database, the expression levels of USP28 were correlated with five mismatch repair (MMR) genes and four methyltransferase genes (DNMT1, DNMT2, DNMT3A, and DNMT3B) across various types of cancer using the Spearman’s correlation method.

### The analysis of USP28 protein localization and PPI network

The Human Protein Atlas (HPA) database (https://www.proteinatlas.org/search) [25] was used to display the distribution of USP28 protein at the subcellular level (U251-MG, U2-OS, and A431 cell lines). The USP28 antibody was purchased from the Sigma-Aldrich company (USA), (1:65). To explore the enrichment of genes related to USP28 in pan-cancer, a protein-protein interaction (PPI) network was created utilizing seven different bioinformatics methods via the GeneMANIA website (http://www.genemania.org).

### Immune cell infiltration analysis in TIMER2

Using the TIMER2 website (http://timer.cistrome.org/), we investigated the association between USP28 mRNA expression and immune cell infiltration across all TCGA tumors [26]. The immune cells include CD8+ T cells, CD4+ T cells, B cells, NK cells, Mast and macrophages cells, cancer-associated fibroblast, endothelial cells, eosinophil, granulocyte monocyte regulatory, hematopoietic stem cells, myeloid dendritic cells, monocytes, plasmacytoid dendritic cells, γ/δ T cells, common lymphoid/myeloid progenitors, and MDSC. Several algorithms were utilized to estimate immune infiltration levels, including XCELL, TIMER, CIBERSORT, CIBERSORT-ABS, MCPCOUNTER, and QUANTISEQ. The results were presented using a heatmap. The purity-adjusted Spearman’s rank correlation test obtained P-values and partial correlation values.

### Gene set enrichment analysis (GSEA)

We downloaded the hallmark gene set “gmt” file (h.all.v7.4.symbols.gmt) from the Molecular Signatures Database (MSigDB) website, which can be accessed at https://www.gsea-msigdb.org/gsea/index.jsp. To identify the biological processes associated with USP28 expression, we performed gene set enrichment analysis (GSEA) using the R tool “clusterProfiler” on differentially expressed genes (DEGs) between low- and high-USP28 expression cancer groups in each cancer type. We calculated each biological process’s normalized enrichment score (NES) and false discovery rate (FDR). The results were visualized in a bubble plot using the R package “ggplot2” [27].

### Prognosis analysis of USP28 in pan-cancer

The prognosis data for overall survival (OS), disease-specific survival (DSS), disease-free interval (DFI), and progression-free interval (PFI) were analyzed using the UCSC Xena database (https://xenabrowser.net/datapages/). We assessed the predictive role of USP28 for specific prognosis types in each tumor by performing univariate Cox regression and the Kaplan-Meier model. We also used bivariate USP28 expression levels to conduct Kaplan Meier curve analysis, and the cutoff was determined using the “surv-cutpoint” function of the “survminer” R package (version 0.4.9). Finally, we displayed the results in a heatmap.

### Correlation between USP28 expression and immune checkpoint genes

The SangerBox website (http://sangerbox.com), an online platform for TCGA data analysis, was employed to explore the connection between USP28 expression and immune checkpoint genes, tumor mutational burden (TMB), microsatellite instability (MSI), neoantigen, and ESTIMATE score in the tumor microenvironment (TME). And the association of gene expression was evaluated using Spearman’s correlation and statistical significance. The ESTIMATE algorithm, developed by Yoshihara et al. to estimate tumor purity in the tumor microenvironment (TME), comprises StromalScore, ImmuneScore, and EstimateScore and is a crucial tool used in this study [28]. Additionally, the immune checkpoint blockade (ICB) therapy cohort was analyzed to test the potential of USP28 to predict immunotherapy response. The VanAllen2015 cohort comprises 42 patients with melanoma cancer who received treatment with CTLA4.

### CNV and methylation profile of USP28 in pan-cancer

We utilized the TCGA methylation module within the UALCAN database to assess the differences in methylation levels of USP28 between tumor and matched normal tissues. In addition, our analysis of the impact of methylation and copy number variation (CNV) on overall survival was conducted using the TIDE website (http://tide.dfci.harvard.edu/query/). The GSCA platform is a web-based tool that integrates multi-omics data using the TCGA database, accessible at http://bioinfo.life.hust.edu.cn/web/GSCA/. The database was also utilized to investigate the correlation between USP28 mRNA expression and copy number variation (CNV) and the extent of USP28 methylation across various tumors.

### Clinical specimen collection

The hepatocellular carcinoma (HCC) tissue samples were collected from inpatients at the First Hospital of Putian City. They were immediately frozen in liquid nitrogen and stored at -80° C until use. The present study received approval from the Medical Ethics Committee of The First Hospital of Putian City. All procedures for sample collection and usage were performed per the approved guidelines. Informed consent was obtained from all patients.

### Cell culture, plasmids construction, and cell transfection

HCC cell lines (HCCLM3, Hep3B, Huh-7, and SK-HEP1) and normal hepatocyte cell line (HL-7702) were obtained from the Cell Bank of Type Culture Collection of the Chinese Academy of Sciences (Shanghai, China). All cells were routinely cultured in Dulbecco’s modified Eagle’s Medium (DMEM, Gibco, USA) supplemented with 10% fetal bovine serum (FBS, Thermo Fisher Scientific, USA) and 1% antibiotics (100 U/ml penicillin and 100 μg/ml streptomycin sulfates, Sigma-Aldrich, USA). The cell lines were cultured at 37° C in a humidified atmosphere with 5% CO2. We obtained small interfering RNA (siRNA) specific to USP28 from Genepharma (Shanghai, China). Next, the HCC cell lines were transfected with siRNA and plasmids by applying Lipofectamine 3000 (Invitrogen, USA) according to the manufacturer’s instructions.

### Western blotting analysis

Total proteins were extracted using Radio-immunoprecipitation (RIPA) assay (Beyotime, Shanghai, China) lysis buffer containing phenylmethanesulfonyl fluoride (Solarbio, Beijing, China). Protein concentration was measured using bicinchoninic acid (BCA) assay kit (Beyotime, Shanghai, China). To begin, the proteins from each sample were separated using an 8% sodium dodecyl sulfate-polyacrylamide gel electrophoresis (SDS-PAGE) method and subsequently transferred to a Polyvinylidene fluoride (PVDF) membrane (Millipore, USA). The following steps were carried out for Western blotting: The membrane was blocked with 5% skimmed milk in Tris-buffered saline tween (Boster, Wuhan, China). Then, the primary antibodies USP28 and Tubulin (Proteintech, USA) were added to the membrane and incubated at 4° C overnight. Subsequently, the membrane was incubated with an HRP-labeled secondary antibody.

### Quantitative real-time PCR (qRT-PCR)

Following the manufacturer’s instructions, we isolated total RNA from tissues and cells using the Trizol reagent (Invitrogen Life Technologies, USA). Subsequently, we performed cDNA synthesis using a TaqMan reverse transcription kit (KR118-03, Tiangen, Beijing, China). Quantitative real-time PCR (qRT-PCR) was performed using an SYBR Green Kit (FP205, Tiangen, Beijing, China). Primer sequences were as follows: USP28 (forward: 5′- GGACCCTTCCTTTCTCCATGA-3’; reverse: 5′-AGGCTGACTGCCTGAGTAATGTC-3′) and GAPDH (forward: 5′-CATACCAGGAAATGAGCTTGAC-3′; reverse: 5′-AACAGCGACACCCACTCCTC-3′). The relative gene expression levels were determined by the 2^–ΔΔCT^ using GAPDH as a reference gene.

### Edu assay and transwell assays

Following the manufacturer’s instructions, we used the Edu cell proliferation assay kit from RiboBio (Guangzhou, China) to measure cell proliferation. The percentage of EdU-positive cells was calculated. For the transwell assays, 1×10^5^ cells were collected and plated in the upper chamber with (invasion) or without (migration) Matrigel (BD Biosciences, USA). The medium containing 20% fetal bovine serum (FBS) was added to the lower. Following a 24-hour incubation period, the non-migrated and non-invading cells on the upper surface of the transwell inserts were removed, and the cells on the lower surface were fixed and stained with 0.1% crystal violet. We then counted the cells in five random microscopic fields and imaged them.

### Immunohistochemical analysis

Immunohistochemical staining was performed by conventional methods. The matched cancerous and normal liver tissue samples were fixed, embedded, sectioned, and deparaffinized. Then, the sections were blocked using serum-free protein block buffer (DAKO, USA) for 30 min. Afterward, they were incubated with anti-USP28 (Proteintech, USA). All sections were observed and captured using a light microscope.

### Statistical analysis

R version 4.0.3 (https://www.r-project.org/) was utilized to conduct all statistical and computational analyses. The Spearman Correlation test is used for bioinformatic validation to assess the link between USP28 expression and targets of interest, such as immune cell infiltration scores, TMB, MSI, MMR genes, methylation transferase genes, CNV, etc. The paired student’s t-test was used to compare the USP28 expression level between tumor and normal tissues. The USP28 expression between groups was evaluated with Kruskal–Wallis test and compared with the Wilcoxon test. To assess the prognostic significance of USP28 expression, univariate Cox regression analysis and Kaplan-Meier method with the log-rank test were performed. The proportions of anti-CTLA4 responders and non-responders were compared between low and high-USP28 cancer subgroups using a chi-square test. A P-value of less than 0.05 was considered statistically significant.

### Data availability statement

The original contributions presented in the study are included in the article. Further inquiries can be directed to the corresponding authors.

## RESULTS

### Clinical landscape of USP28 expression levels in pan-cancer

**Figure 1:**
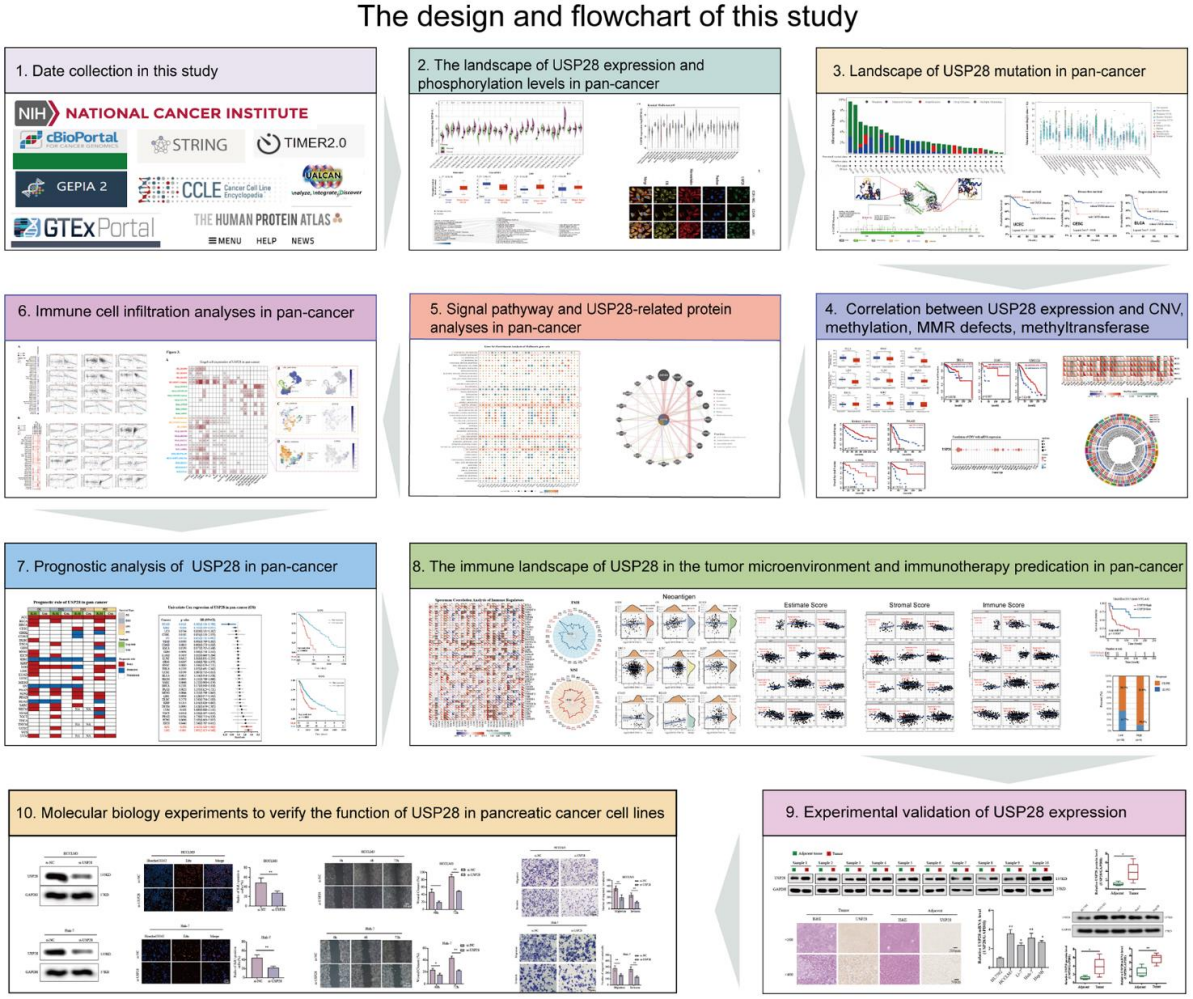
**The design and flowchart of this study.** *p < 0.05; ** *p* < 0.01; *** *p* < 0.001.

We used the public databases to conduct pan-cancer analysis of USP28, including the landscape of expression, genetic alteration, methylation, MMR defects, CNV, relevant signal pathways, immune cell infiltration, a correlation between expression and survival, immune landscape, and immunotherapy predication. Figure 1 summarizes the flowchart of this pan-cancer analysis, which was carried out to investigate the functions and potential mechanisms of USP28 in the pathogenesis or clinical prognosis of different cancers.

**Figure 2:**
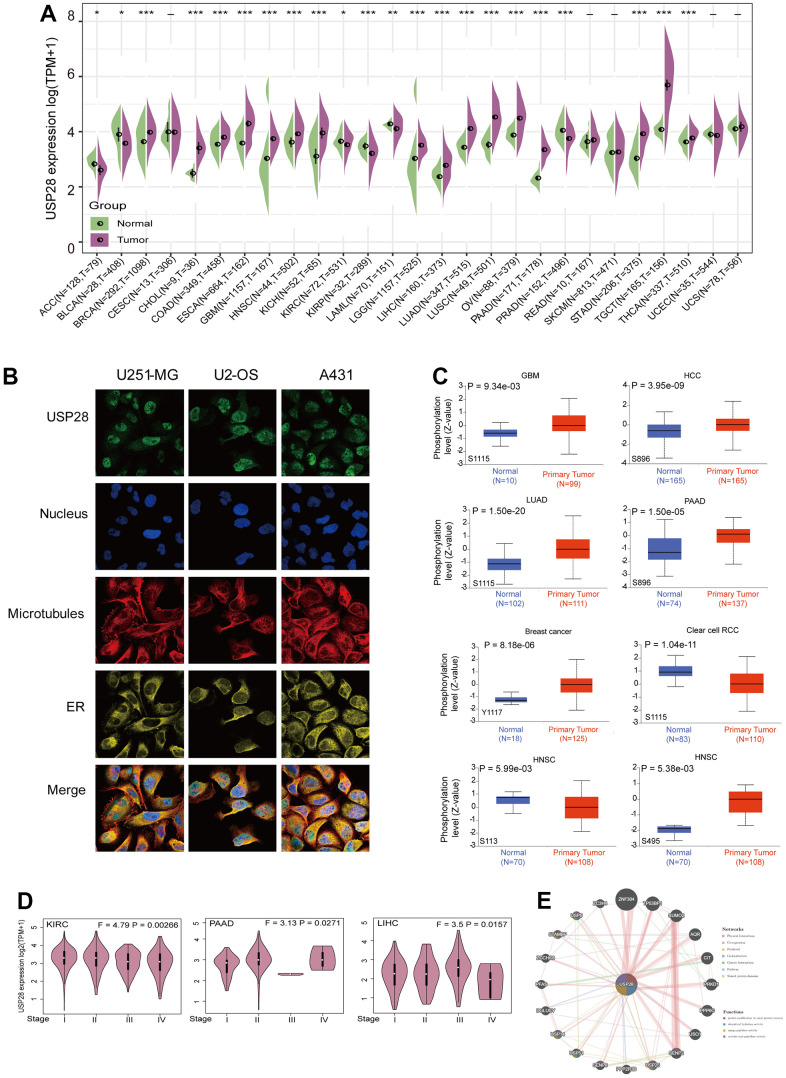
**Clinical landscape of USP28 expression levels in pan-cancer.** (**A**) The difference in USP28 expression between tumor and normal tissues in different cancers through TCGA and GTEx datasets. (**B**) The immunofluorescence images of the USP28 protein, nucleus, endoplasmic reticulum (ER), microtubules, and the merged images in U251-MG, U2-OS, and A431 cell lines. (**C**) Based on the CPTAC dataset, the expression level of USP28 phosphoprotein, including Y1117, S1115, S896, S113, and S495, between normal tissue and primary tissue of selected tumors via the UALCAN. (**D**) The correlation of USP28 expression levels with pathological stages (stage I stage II, stage III, stage IV) was analyzed using the TCGA dataset. Log2 (TPM+1) was applied for the log scale. (**E**) PPI network to identify the USP28-interacting proteins using the GeneMANIA database. *p < 0.05; ** *p* < 0.01; *** *p* < 0.001.

We first characterized the mRNA expression of USP28 in different normal human tissues through the GTEx database. As shown in Supplementary Figure 1A, USP28 expression levels varied in various tissues, which was highest in muscle tissue compared with other tissues. And mRNA expression levels of USP28 varied significantly in 22 cancer cell lines according to the CCLE database (Supplementary Figure 1B). Furthermore, we evaluated the expression status of USP28 in various cancers and normal tissues by TCGA and GTEx databases. USP28 was highly expressed in BRCA, CHOL, COAD, ESCA, GBM, HNSC, KICH, LGG, LIHC, LUAD, LUSC, OV, PAAD, STAD, TGCT, and THCA compared with their adjacent normal tissues. A low USP28 expression level was observed in ACC, BLCA, KIRC, KIRP, LAML, and PRAD. However, we did not find a significant difference for other tumors, such as CESC, READ, SKCM, UCEC, and UCS (Figure 2A). Additionally, based on the HPA website, immunofluorescence images revealed that the USP28 protein was predominantly localized and distributed in the nucleus of U251-MG, U2-OS, and A431 tumor cell lines (Figure 2B). Finally, we identified the involvement of USP28 in 13 diseases based on the OPEN TARGET platform, such as cancer or begin tumor, nutritional or metabolic disease, and gastrointestinal disease (Supplementary Figure 1C). Therefore, the above results indicate that USP28 is abnormally expressed in various tumors and is closely related to multiple diseases.

### Analysis of USP28 phosphorylation levels

We compared the differences in USP28 phosphorylation levels between primary tumors and normal tissues by the CPTAC dataset. Seven types of tumors were explored, including Clear cell RCC, GBM, HCC, HNSC, LUAD, BRCA, and PAAD. The phosphorylation levels of USP28 in different sites existed for certain differences in various tumors (Figure 2C). Specifically, the Y1117 of USP28 exhibited a higher phosphorylation level in breast cancer than in normal tissues. And the phosphorylation levels of S1115 were increased in GBM and LUAD, while they were decreased in Clear cell RCC. The phosphorylation levels of S896 also increased in HCC and PAAD. However, in HNSC, the phosphorylation level of S113 was decreased, whereas the phosphorylation levels of S495 were increased. Next, with the HEPIA2 database support, we exhibited a significant correlation between USP28 expression and the pathological stages of some cancers, including KIRC, PAAD, and LIHC (Figure 2D) but not others (Supplementary Figure 1D). Finally, the PPI network of USP28 was created by the GeneMANIA online platform. The findings revealed a robust physical association between USP28 and ZNF304, a key player in cancer metastasis [29]. The study found that the stabilization of ZNF304 by USP28 results in the hypermethylation and transcriptional silencing of tumor-suppressor genes during oncogenic transformation [30], consistent with the results of the physical interactions (Figure 2E).

### Single-cell analysis of USP28 in pan-cancer

**Figure 3:**
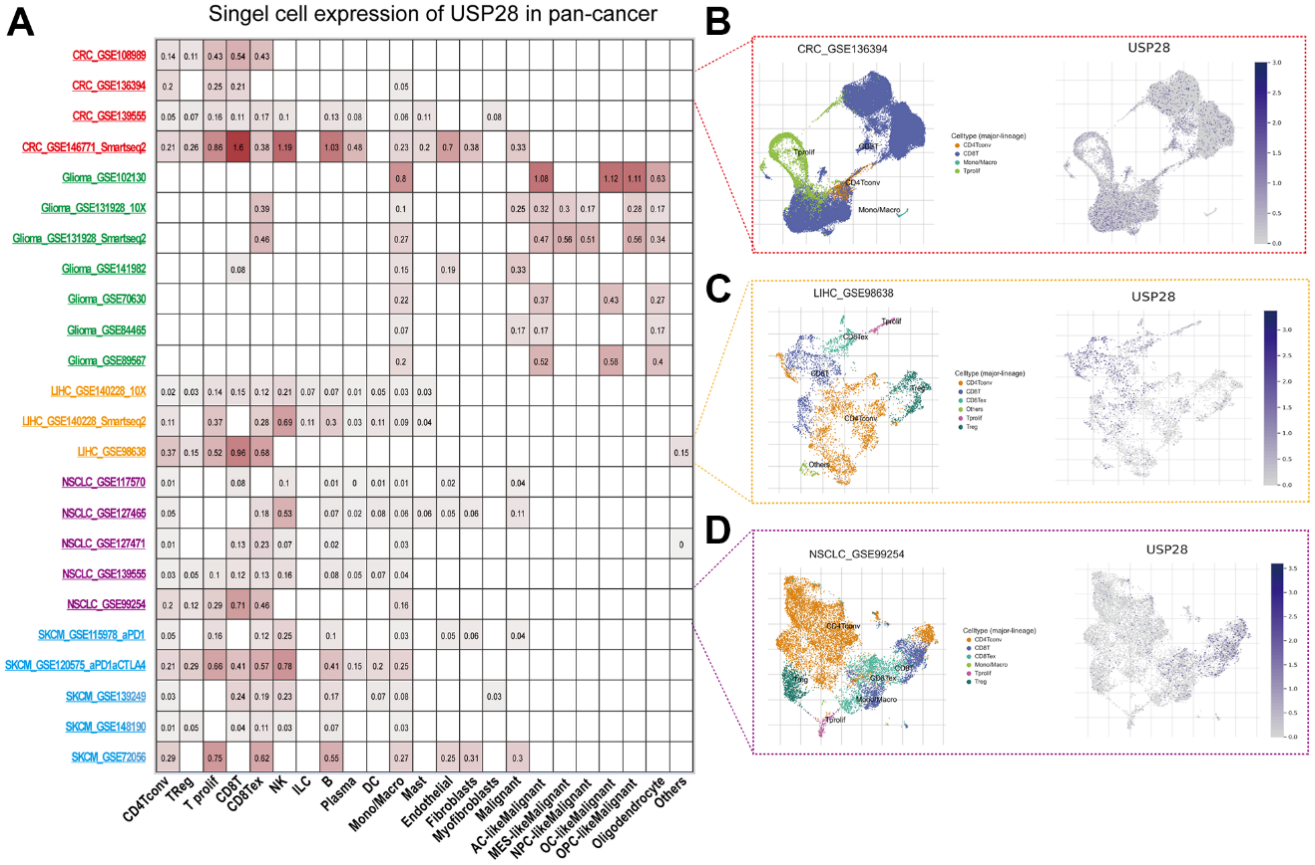
**Single-cell analysis of USP28 in pan-cancer.** (**A**) Summary of USP28 in Single-cell datasets. (**B**) The Scatter plot showed the distributions and USP28 expression of 4 different cell types of the GSE136394 CRC dataset. (**C**) The Scatter plot showed the distributions and USP28 expression of 6 different cell types of the GSE986638 LIHC dataset. (**D**) The Scatter plot showed the distributions and USP28 expression of 6 different cell types of the GSE99254 NSCLC dataset.

We performed the single-cell analysis of USP28 in single-cell datasets of cancer samples to understand the main cell types that express the USP28 in cancer microenvironments using the TISCH web tool. The heatmap shows that USP28 was mainly expressed in the immune cells (Figure 3A). In the GSE136394 and GSE98638 datasets, USP28 expression is primarily expressed in T cells in the CRC and LIHC microenvironment (Figure 3B, 3C). In the GSE99254 NSCLC dataset, USP28 is highly expressed in CD8T cells (Figure 3D). Our results suggest that the immune expression of USP28 in different tumors is somewhat different.

### Mutation landscape of USP28 in pan-cancer

**Figure 4:**
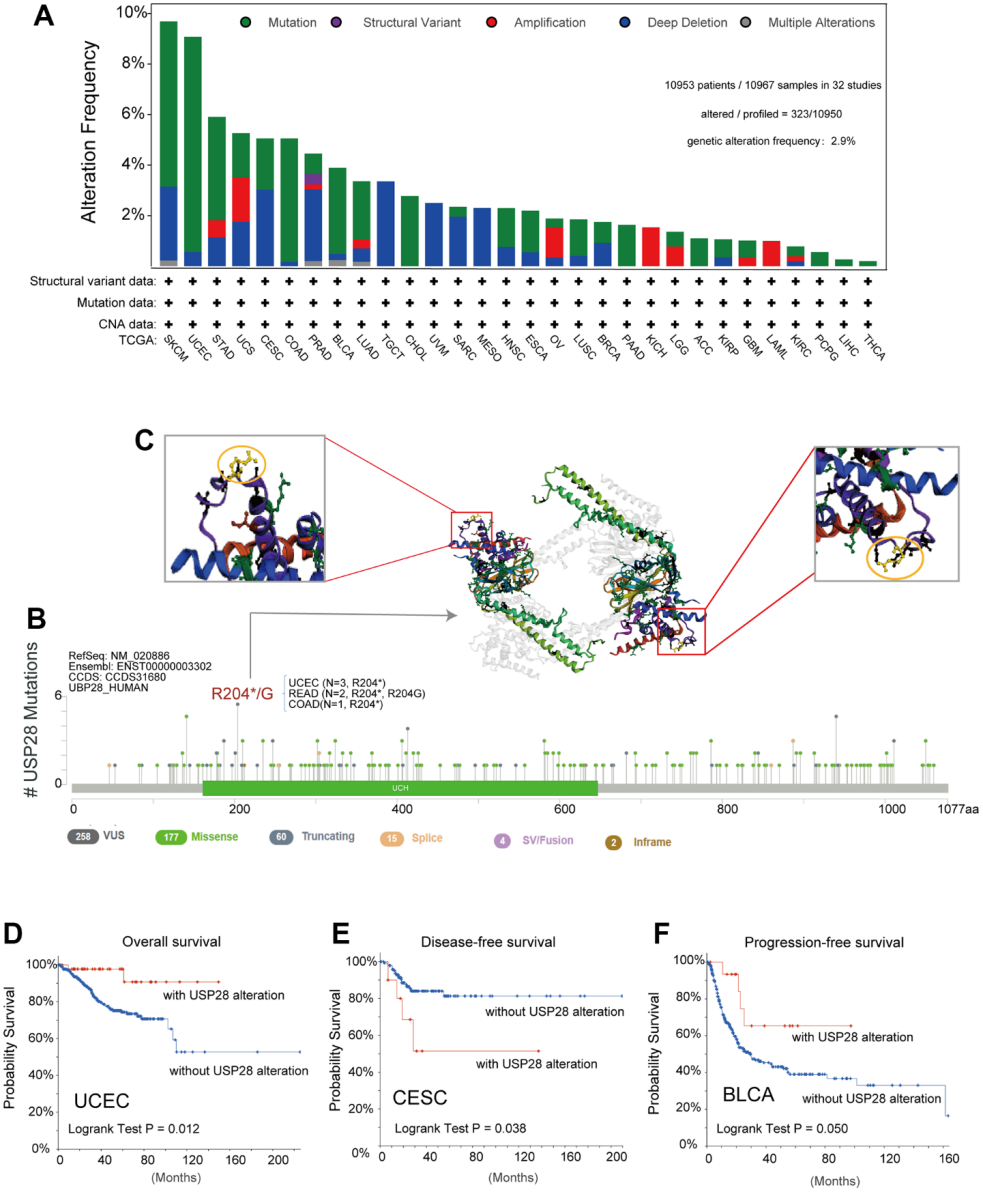
**The landscape of genetic alterations of USP28 in pan-cancer.** (**A**) The alteration frequency with the mutation type of USP28 for the TCGA tumors was analyzed by the cBioPortal tool. (**B**) The protein domain displayed all the mutation sites and mutation types of USP28. (**C**) The highest alteration frequency (R204*/G) was shown in the 3D structure of USP28 (labeled in yellow). (**D**–**F**) The potential correlation between alteration status of USP28 and clinical prognostic indices, including overall survival, disease-free survival, and progression-free survival in specific cancers.

Considering the aberrant expression of USP28 in various cancers, we further observed the genetic alteration status of USP28 across multiple tumor samples of TCGA cohorts. The mutation counts of each type in different cancers, including not mutated, deep deletion, missense, shallow deletion, truncating, gain in a frame, diploid, splice, amplification, and structural variant, were exhibited in Supplementary Figure 2A. In-depth, as shown in Figure 4A, the highest alteration frequency of USP28 (>9%) appeared in SKCM tumors with “mutation” and “deep deletion” as the primary types. The second highest alteration frequency at nearly 9% occurs in UCEC, with “mutation” as the primary type. The “amplification” type was the only type in the KICH, and LAML tumors showed an alteration frequency below 2%. In addition, “mutation” is the only type in CHOL, PAAD, ACC, PCPG, LIHC, and THCA tumors. It is worth noting that the “structural variant” only appeared in the PRAD tumor.

Moreover, we also provided information about the specific locations and frequency of alterations in the USP28 gene across different cancer types (Figure 4B and Supplementary Table 2). The missense mutation was the primary type of genetic alteration, and R204*/G in the UCH domain, which was detected in three cases of UCEC, two cases of READ, and one case of COAD tumors, can induce a truncating mutation, translation from Arginine (R) to stop codon or Glycine (G) at 204 sites of USP28 protein. Subsequently, the 3D structure of R204*/G in USP28 was observed in Figure 4C. Importantly, we also explored the potential association between the genetic alteration of USP28 and the clinical survival prognosis of cases in some cancers. The results indicated that UCEC cases with USP28 alteration showed a better prognosis in overall survival (Figure 4D) than cases without USP28 alteration. However, there was no significant difference in disease-specific, disease-free, and progression-free survival (Supplementary Figure 2B). Moreover, CESC cases with USP28 alteration had lower disease-free survival (Figure 4E), and BLCA cases with USP28 alteration had a better prognosis in progression-free survival (Figure 4F).

### Correlation analysis with methylation profile, CNV, and MMR defects

**Figure 5:**
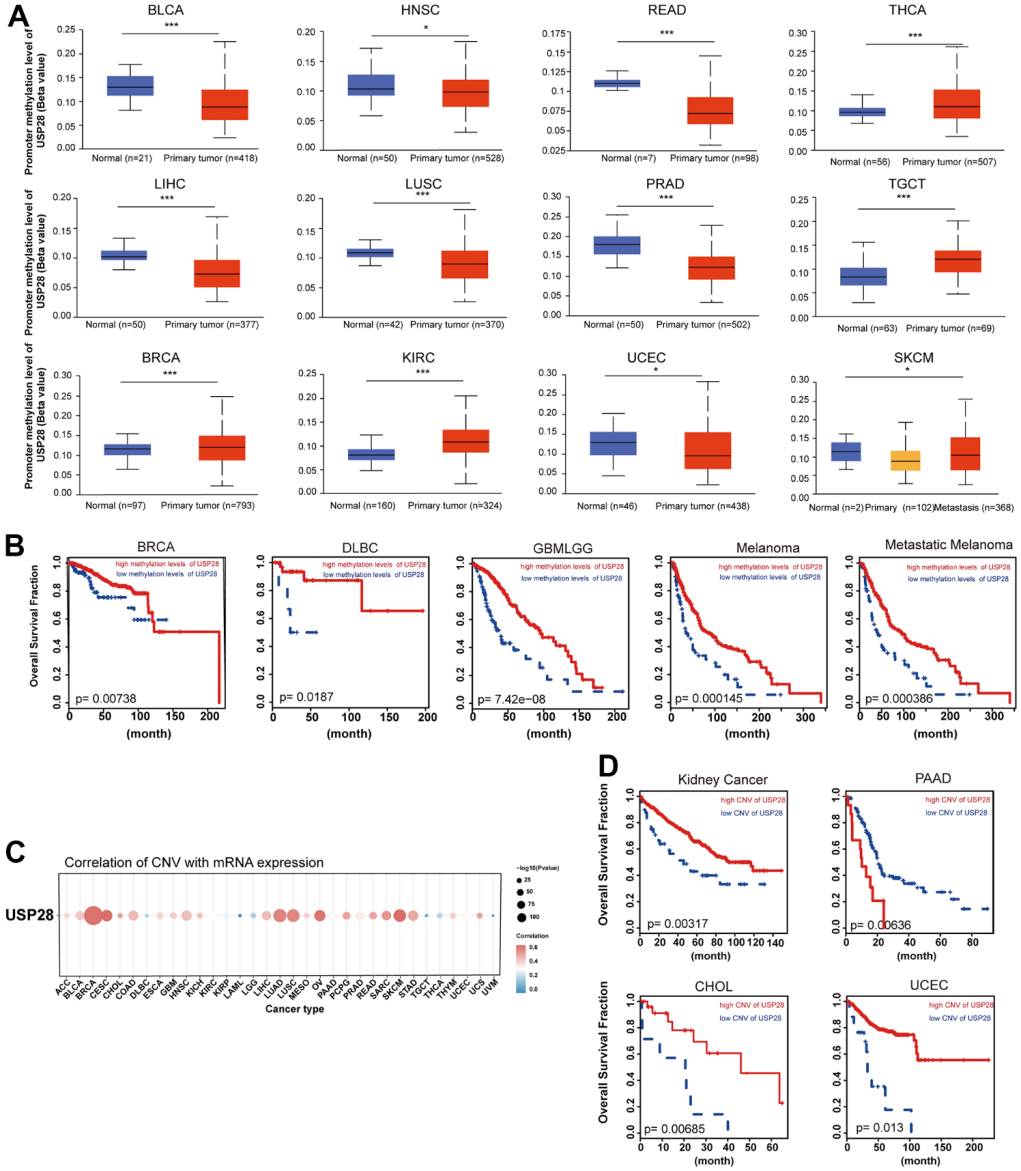
**Correlation and prognosis analysis with methylation profile and CNV.** (**A**) Boxplots showing differential USP28 methylation levels between tumor and adjacent normal tissues across the TCGA database. (**B**) Kaplan-Meier curves of overall survival differences between TCGA cancer cohorts with high methylation levels and those with low methylation levels of USP28. Only TCGA cancers with statistically significant differences between cohorts were presented. (**C**) The heatmap exhibiting association between USP28 CNV and mRNA expression in various cancers. (**D**) Kaplan-Meier curves of overall survival differences between TCGA cancer cohorts with high CNV levels and those with low CNV levels of USP28. *p < 0.05; ** *p* < 0.01; *** *p* < 0.001.

To elucidate the possible involvement of USP28 in tumor progression, we investigated the correlation between USP28 expression and mutations in mismatch DNA repair (MMR) genes. USP28 expression was significantly correlated with the five MMR genes in all cancers (Supplementary Figure 2C). Next, the USP28 methylation landscape was also analyzed. The promoter methylation level of USP28 was significantly decreased in BLCA, HNSC, READ, LIHC, LUSC, SKCM, UCEC, and PRAD. In contrast, the increased promoter methylation levels of USP28 were observed in BRCA, KIRC, TGCT, and THCA (Figure 5A). These findings indicated that USP28 methylation is significantly associated with mRNA levels in various cancers. Subsequently, we further evaluated the influence of USP28 methylation status on prognosis in multiple cancers. Importantly, we found hypermethylation of USP28 was positively associated with higher overall survival in DLBC, GMBLGG, Melanoma, and Metastatic Melanoma cases. In contrast, hypomethylation of USP28 was associated with a good prognosis in BRCA (Figure 5B). Given that DNA methylation is the covalent bonding of a methyl group at the 5’ carbon position of cytosine in genomic CpG dinucleotides by DNA methyltransferases [31], the relationship between DNA methyltransferases and USP28 expression was also assessed. The data suggested that USP28 expression is strongly related to all cancers’ four types of DNA methyltransferases (Supplementary Figure 2D).

Additionally, we explored the association between USP28 mRNA and copy number variation (CNV) through Spearman’s analysis. A substantial positive connection exists between USP28 mRNA expression and CNV in BRCA, CESC, LUAD, LUSC, OV, and SKCM (Figure 5C). Therefore, we explored the consequences of USP28 CNV status in various cancer. As shown in Figure 5D, high CNV of USP28 was associated with better overall survival in Kidney Cancer, CHOL, and UCEC. Conversely, a high CNV of USP28 could lead to lower overall survival in PAAD. Taken together, these results suggest that USP28 could potentially modulate tumorigenesis and cancer progression by exerting control over the epigenetic state of cancer cells.

### Gene set enrichment analysis of USP28 in pan-cancer

**Figure 6:**
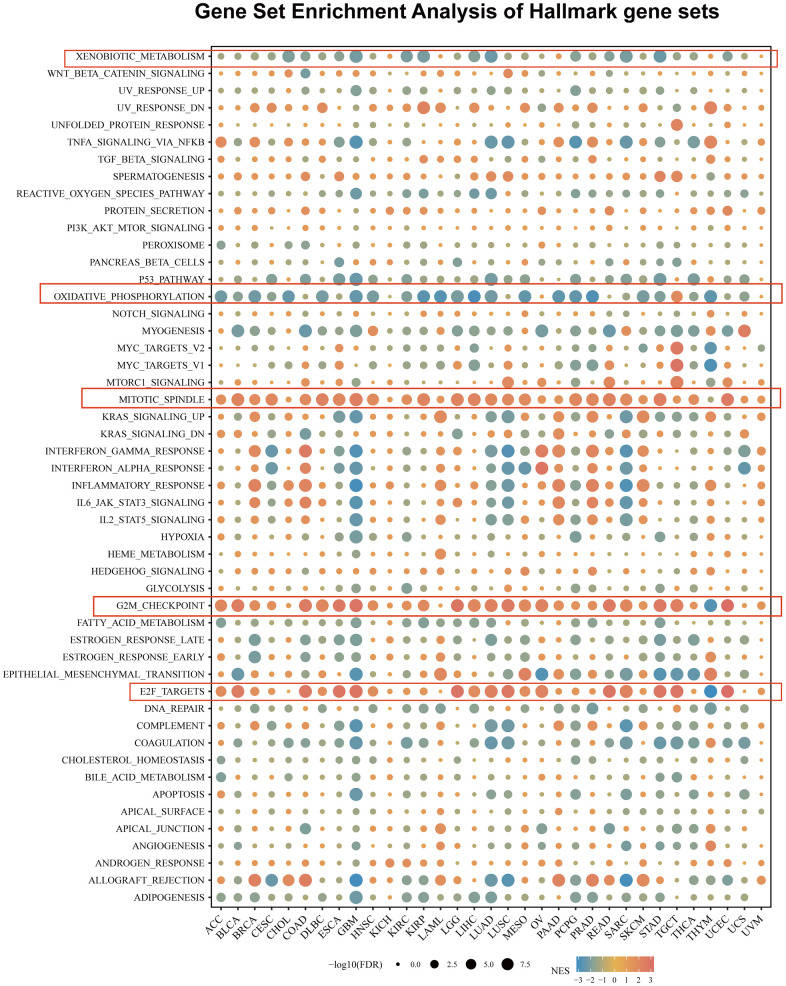
**The hallmarks gene set enrichment analysis (GSEA) of USP28 in pan-cancer.** The circle size represents the FDR value of the enriching term in each cancer, and the color indicates each term’s normalized enrichment score (NES).

To investigate the biological processes associated with USP28 expression in pan-cancer, we conducted differential expression analysis between the top 30% and bottom USP28 expression subgroups in each cancer type. The differentially expressed genes (DEGs) in each cancer type are presented in Supplementary Table 3. Subsequently, the GSEA was performed on the DEGs in pan-cancer to determine the USP28-associated cancer hallmarks. The results revealed that the expression level of USP28 was closely related to immune-related signaling pathways, such as xenobiotic metabolism, oxidative phosphorylation, mitotic spindle, G2 checkpoint, and E2F targets pathways, especially in GBM, LUSC, LUAD, SARC, and UCEC. In addition, the USP28 expression of GBM tissue was negatively related to most signal pathways, including xenobiotic metabolism, TNFA-signaling-via-NFKB, P53 pathway, oxidative phosphorylation, KRAS signaling, inflammatory-response, IL6-JAK-STAT3-signaling, complement, coagulation, and apoptosis. It was positively related to the mitotic spindle, G2 checkpoint, and E2F target pathways (Figure 6). Taken together, the above results indicate that the expression of USP28 is associated with the immune activation status of cancer. This provides some reference directions for further research on the role of USP28 in cancer occurrence and progression.

### Immune infiltration analyses of USP28 in pan-cancer

**Figure 7:**
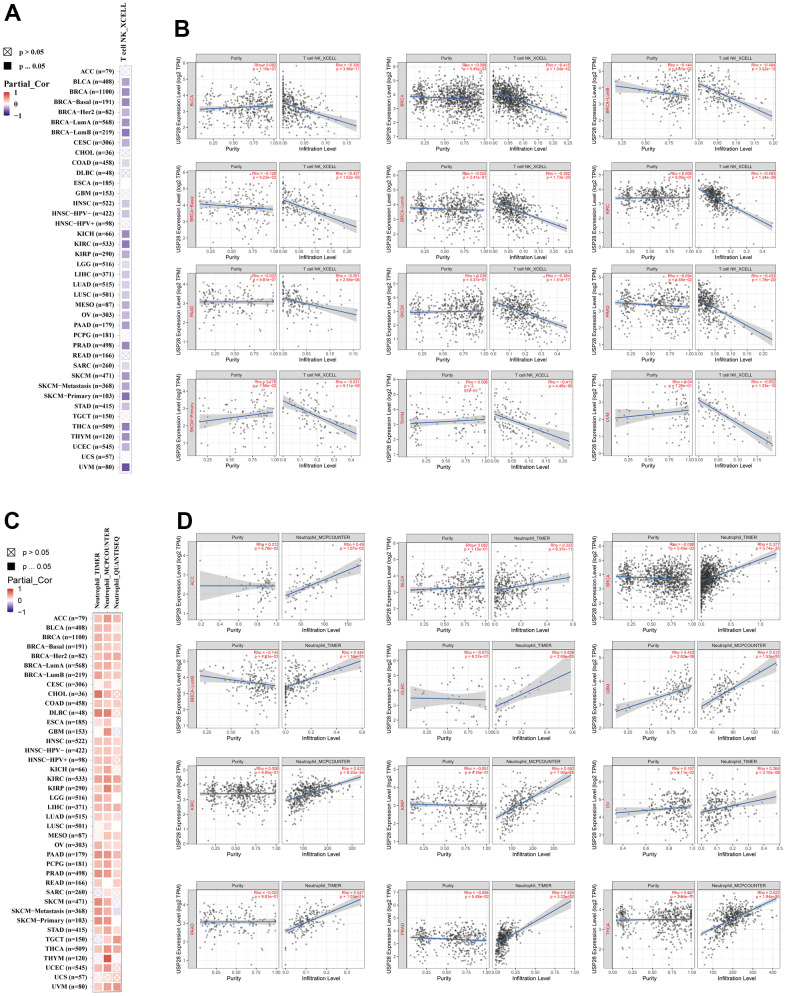
**Correlation analysis between USP28 expression and cell infiltration of cancer-associated fibroblast.** The potential connection between the expression level of the USP28 gene and the infiltration level of T cell NK (**A**, **B**) and neutrophil (**C**, **D**) was used to explore based on different algorithms in the TIMER database.

Cancer’s presence, progression, or metastasis is closely linked to the infiltration of immune cells into tumor tissue [32]. Therefore, we explore the relationship between USP28 and immune cell infiltrations by the TIMER2 database (Figure 7). After a series of analyses, we observed a statistically negative correlation between T cell NK and USP28 expression in most cancers based on the XCELL algorithm. Moreover, according to the TIMER, MCPCOUNTER, and QUANTISEQ algorithms, we observed a significant positive correlation between USP28 expression and neutrophil’s estimated infiltration value in the pan-cancer analysis. In addition, multiple algorithm results showed that the expression of USP28 was positively correlated with the infiltration levels of CAFs, B cells, and macrophages in most cancers (Supplementary Figure 3). The above findings indicate that USP28 may impact cancer progression, prognosis, and treatment by interacting with immune cells. To better analyze the immune aspects of USP28 in pan-cancer, we calculated the correlation between USP28 levels and EstimateScore (Supplementary Figure 4), ImmuneScore (Supplementary Figure 5), and StromalScore (Supplementary Figure 6). As shown in Supplementary Figure 7, the top three tumors with a significant correlation between USP28 and StromalScore were SARC (R = -0.499, P < 0.001), GBM (R = -0.462, P < 0.001), and TGCT (R = -0.393, P < 0.001); The top three tumors whose USP28 expression was most significantly correlated with ImmuneScore were SARC (R = -0.438, P < 0.001), GBM (R = -0.511, P < 0.001), and UCEC (R = -0.263, P < 0.001); The top three tumors with the most significant relationship between USP28 expression and EstimateScores were SARC (R = -0.486, P < 0.001), GBM (R = -0.506, P < 0.001), and LUCS (R = -0.252, P < 0.001). We also analyzed the relationship between USP28 expression and neoantigens in pan-cancer (Supplementary Figure 8). Taken collectively, these findings suggest a broad association between USP28 expression and immunity across various types of cancer.

### Relationships between USP28 and immune regulators, TMB, and MSI

**Figure 8:**
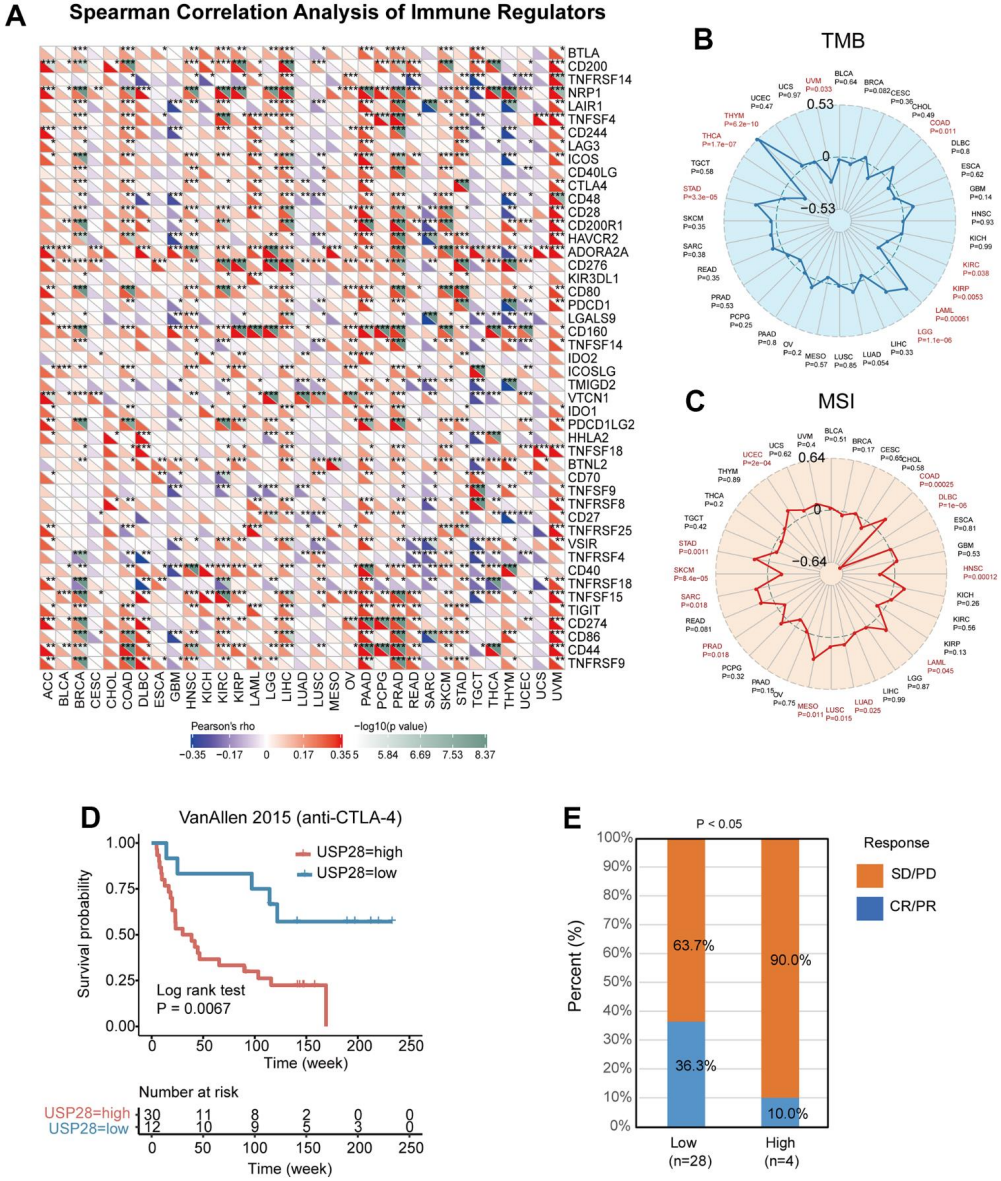
**Relationships between USP28 and immune checkpoint genes, TMB, and MSI.** (**A**) Heatmap exhibiting the correlation between USP28 and immune checkpoint gene expression in 33 cancer types from the TCGA database. Spearman’s rank correlation coefficient was used. (**B**) The association analysis between USP28 expression and tumor mutation burden (TMB) in pan-cancer. (**C**) The correlation analysis between USP28 expression and microsatellite instability (MSI) in pan-cancer was described. (**D**) Survival analysis of patients with high (n = 30) and low (n = 12) USP28 expression based on OS data from patients receiving anti-CTLA-4 immunotherapy, (**E**) and proportions of patients with different therapeutic responses *p < 0.05; ** *p* < 0.01; *** *p* < 0.001.

Given the link between USP28 expression and immune infiltration, we looked into the relationship between USP28 expression and immune checkpoint gene expression. We found a strong positive relationship between USP28 and most immune checkpoint genes in ACC, BRCA, LIHC, PAAD, PRAD, SKCM, and UVM. And USP28 had a negative association with some immune checkpoint genes in GBM, SARC, TGCT, and THYM tumors. In most TCGA cancers, except for SARC and TGCT, there was a strong positive correlation between USP28 and CD276 and Neuropilin-1 (NRP1) (Figure 8A). NRP1 was closely associated with a variety of genes in pan-cancer studies, such as IGF-1 [33], PDIA3 [34], and CD36 [35].

The correlation between USP28 expression and TMB and MSI was analyzed to understand the role of USP28 in predicting the efficacy of immune checkpoint inhibitors (ICIs) [36]. As shown in Figure 8B, for the correlation between USP28 expression and TMB, positive associations were discovered in THYM, STAD, LGG, LAML, and COAD. Negative correlations were found in UVM, THCA, KIRP, and KIRC. Moreover, positive correlations with MSI were identified in UCEC, STAD, SARC, MESO, LUSC, LUAD, LAML, and COAD, and negative correlations with SKCM, PRAD, HNSC, and DLBC (Figure 8C). The results suggest that USP28 expression levels may serve as a predictive biomarker for the efficacy of immune checkpoint inhibitors in the corresponding cancers. Next, we investigated the potential of USP28 as a predictor of cancer immunotherapy response. As depicted in Figure 8D, the relationship between USP28 and anti-CTLA4 therapy response in patients with melanoma tumors revealed that low-expression USP28 patients outlived high-expression patients in terms of survival rate and time. In the VanAllen 2015 cohort of melanoma tumors, patients with high USP28 expression responded 10% to anti-CTLA4 therapy, which was significantly lower than the 36.3% response rate observed in low-USP28 expression patients (Figure 8E).

### Clinical prognostic significance of USP28 in pan-cancer

**Figure 9:**
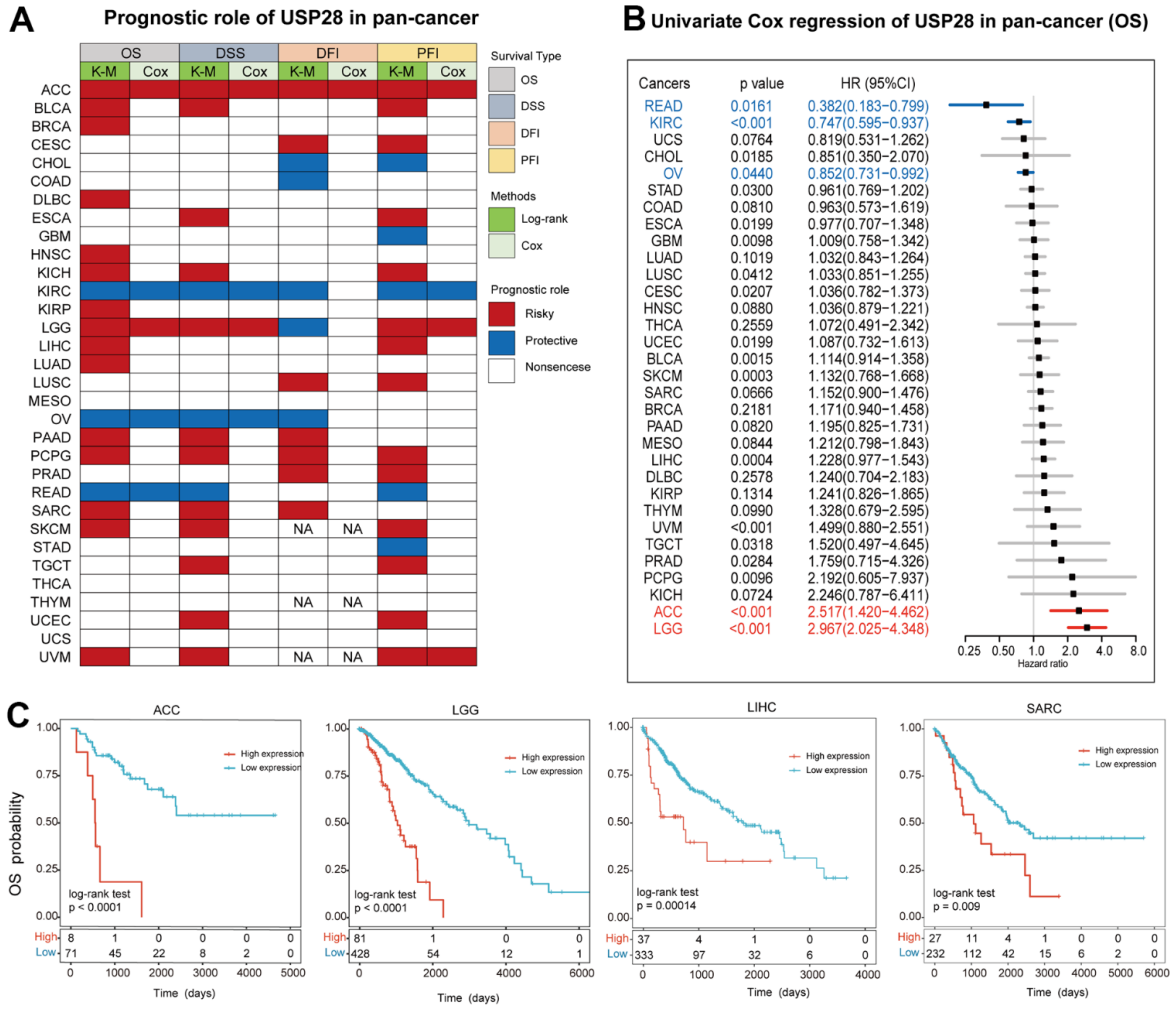
**Prognostic analysis of USP28 in pan-cancer.** (**A**) The heatmap described the correlation between USP28 expression levels and overall survival (OS), disease-specific survival (DSS), disease-free interval (DFI), and progression-free interval (PFI) using the univariate Cox regression and Kaplan-Meier models. (**B**) The forest plot described the prognostic role of USP28 in pan-cancer. (**C**) Kaplan-Meier overall survival curves of USP28 in ACC, LGG, LIHC, and SARC. *p < 0.05; ** *p* < 0.01; *** *p* < 0.001.

To explore the potential prognostic value of USP28 in different types of cancer, we analyzed four prognostic indicators using Kaplan-Meier and univariate Cox regression methods. The heatmap showed the relationship between USP28 expression and four prognoses (Figure 9A). USP28 expression was significantly related to the prognosis of most cancers except MESO, THCA, UCS, and THYM. Specifically, the OS analysis results showed that USP28 was a risk factor for poor prognosis of ACC, BLCA, BRCA, DLBC, HNSC, KICH, KIRP, LGG, LIHC, LUAD, PAAD, PCPG, SARC, SKCM, and UVM while a protective factor for patients with KIRC, OV, and READ. It should be noted that USP28 was identified as a risk factor associated with poor prognosis in ACC and PCPG, as it was significantly correlated with four different prognostic survival indicators in these cancers. Conversely, in KIRC tumors, USP28 was a protective factor for four different predictive types, based on results from a log-rank test statistical analysis. Using univariate Cox regression, the results of the forest plot demonstrated that the downregulation of USP28 expression was associated with a delay in overall survival (OS) time. (Figure 9B): ACC (HR = 2.517 [95% CI, 1.420 – 4.462], P < 0.001), LGG (HR = 2.967 [95% CI, 2.025 – 4.348], P < 0.001). The upregulation of USP28 expression was related to the time delay of OS: READ (HR = 0.382 [95% CI, 0.183 – 0.799], P = 0.0161), KIRC (HR = 0.747 [95% CI, 0.595 – 0.937], P < 0.001), and OV (HR = 0.852 [95% CI, 0.731 – 0.992], P = 0.0440). Several studies indicate that USP28 is closely related to the progression and prognosis of liver carcinogenesis [37], GBM [38], and sarcoma [39], so we performed Kaplan–Meier curves analysis of ACC, LGG, KICH, and SARC, which indicated that a higher USP28 was associated with poor OS (Figure 9C) outcomes. Hence, the prognostic role of USP28 in predicting cancer prognosis suggests that further investigation is needed better to understand the function of USP28 in cancer cells.

### Interfering with the expression of USP28 inhibited cell lines proliferation, migration, and invasion

**Figure 10:**
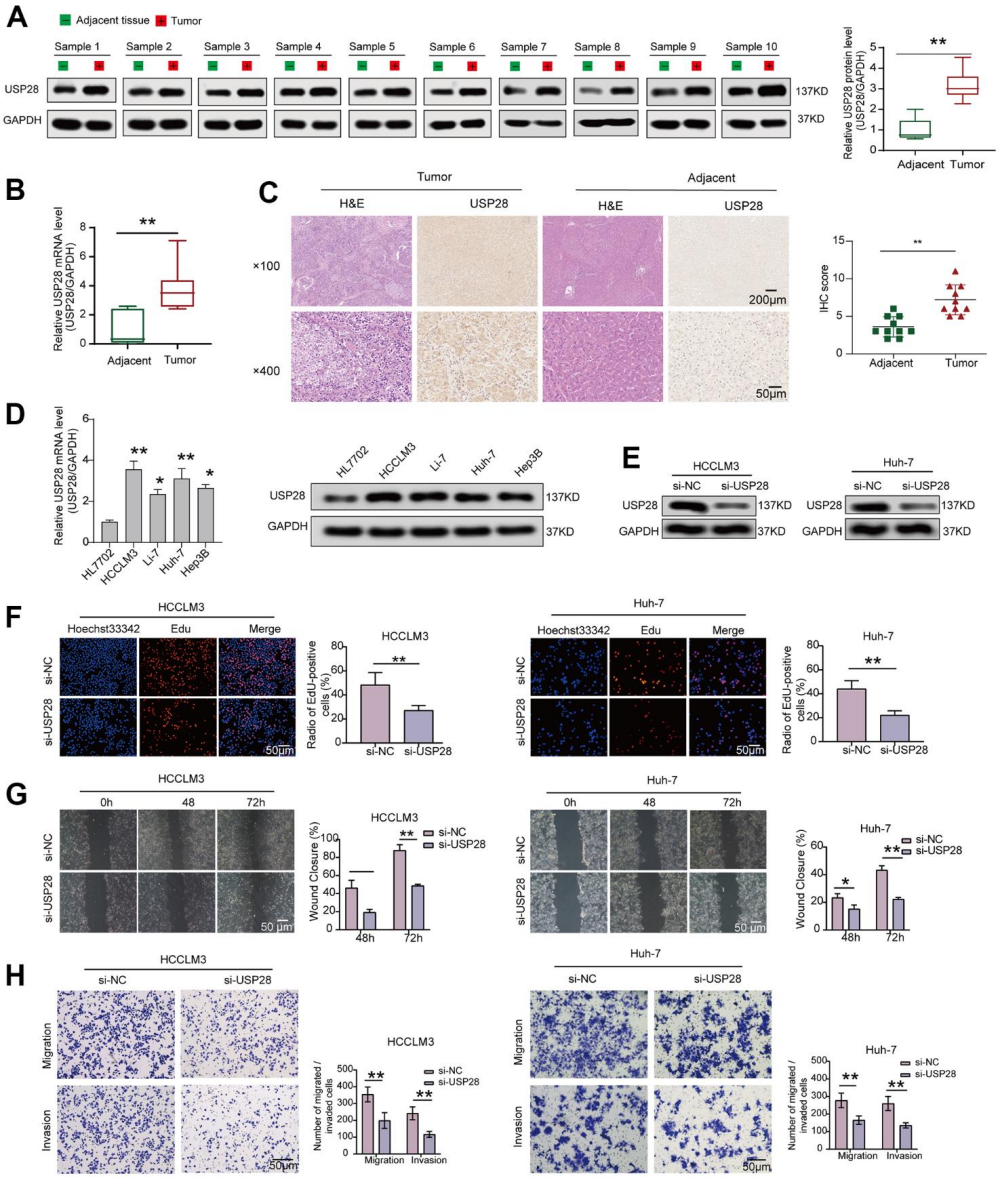
**Interfering with the expression of USP28 inhibited cell lines proliferation, migration, and invasion.** (**A**) The protein level of USP28 in HCC and normal tissues. (**B**) Relative mRNA expression of USP28 in HCC tissues compared to normal tissues. (**C**) Immunohistochemical staining of USP28 in HCC tissue and adjacent tissue. (**D**) qPCR and Western blotting analysis of USP28 mRNA and protein expression in four HCC cell lines (HCCLM3, Li-7, Huh-7, Hep3B) and normal liver cell line (HL7702). GAPDH was used as an internal control. (**E**) The efficiency of USP28 siRNA (si-USP28) in HCCLM3 and Hep3B was confirmed by Western blotting. (**F**) EdU assays for HCCLM3 and Huh-7 were performed to evaluate cell proliferation ability after transfecting si-USP28. (**G**, **H**) Scratch wound healing assay and transwell assays assessed the migration and invasion abilities in HCCLM3 and Huh-7 cells. (Original magnification, ×200; scale bars, 50 μm). *p < 0.05; ** *p* < 0.01; *** *p* < 0.001.

The results, as mentioned above, have pinpointed the potent roles of USP28 across tumor types, especially in HCC. Liver cancer is one of the most common cancers and a major cause of cancer deaths in China, which accounts for over 50% of new cases and deaths worldwide [40]. As a result, we concentrated on the HCC to explore the expression and biological roles of USP28 using the clinical samples and HCC cell lines. As shown in Figure 10A, USP28 protein expression was significantly increased in HCC tissues. In keeping with the increased USP28 protein, qRT-PCR data indicated that the mRNA expression level of USP28 in tumor tissues was higher than in adjacent tissues (Figure 10B). Similarly, IHC staining results suggested that the expression of USP28 was upregulated in the HCC tissues compared with the corresponding normal tissues (Figure 10C). Moreover, WB and qRT-PCR results also suggested that USP28 expression in the HCC cell lines (HCCLM3, Hep3B, Li-7, Huh-7) was higher than that in normal liver cells (HL7702) (Figure 10D). These findings suggest that USP28 expression was upregulated in HCC tissues and cell lines, consistent with the public database analysis. Lastly, some molecular biology experiments were used to explore the role of USP28 on tumorigenesis further. USP28-targeting siRNA vectors downregulated USP28 in HCCLM3, and Huh-7 (Figure 10E). As illustrated in Figure 10F, EdU staining assays showed that knocking down USP28 inhibited cell proliferation. Subsequently, cell scratch (Figure 10G) and transwell assays (Figure 10H) were performed to assess the impact of USP28 on cell migration and invasion. The results revealed that the knockdown of USP28 also dramatically decreased the migration and invasion of HCCLM3 and Huh-7. These findings support the notion that USP28 plays a significant oncogenic role in enhancing cell proliferation, migration, and invasion.

## DISCUSSION

USP28, as a critical member of a family of deubiquitinating enzymes, is involved in many physiological and pathological progress of cancers, including physiological homeostasis of the ubiquitination process, DNA-damage response, apoptosis, cancer migration, differentiation [6, 41–43]. Accumulating evidence suggested that USP28 was involved in multiple-pathway. One study found that USP28 could affect the cell cycle and proliferation by regulating MYC abundance in colon and breast carcinomas [44]. Moreover, USP28 was the regulator of DNA-damage response for acting a critical role in DNA-damage-induced ubiquitination and deubiquitination [41]. Further, the high expression of the deubiquitinating enzyme USP28 was targeted by miR-4295, promoting non-small cell lung cancer cell proliferation [45]. However, most cancers’ clinical translational potential and immune signaling pathways remain unknown.

Furthermore, the heterogeneity of the tumor microenvironment in cancer patients means that immunotherapy is only effective for a subset of patients with cancer. Therefore, biomarkers that accurately predict the patient’s response to immunotherapy will be very important in improving the individualized immunotherapy of cancer patients. After a thorough literature search, we could not locate any publications that performed a pan-cancer analysis of USP28 across different tumor types. Thus, the USP28 gene in pan-cancer was comprehensively examined, including gene expression, genetic alteration, DNA methylation, signal pathway, protein phosphorylation, immune cell infiltration, and relationships of immune regulators. In total, USP28 is a reliable and valuable prognostic biomarker in many tumors.

We first analyzed the USP28 mRNA expression in the normal and cancer tissues using TCGA and GTEx datasets. The results revealed that USP28 was highly expressed in most cancers, including LIHC. Furthermore, our molecular biology experiments demonstrated that the expression of USP28 was markedly elevated in clinical hepatocellular carcinoma (HCC) tissues compared to adjacent normal tissues, corroborating the findings obtained through database analysis. The conclusion of this study indicated a high phosphorylation level of USP28 in some primary tumors compared with normal tissues. Some studies have reported that phosphorylation of USP28 was closely related to the progression of cancer [6, 46]. The latest research has found that ATR phosphorylates USP28 (S67 and S714) and increases its enzymatic activity, further confirming that targeting the USP28-Np63 axis in sensitizing squamous cell tones down this DNA damage response pathways [46]. Furthermore, this study found that the phosphorylation levels of USP28 in HNSC showed opposite expression trends at S113 and S495. However, whether the above two USP28 phosphorylation sites have functional significance in tumor development, the clinical importance of these post-translational modification sites remains to be further investigated.

Gene mutation and methylation can regulate gene expression [47, 48], the primary cause of tumorigenesis [49]. We first found that USP28 expression was strongly correlated with CNV in some cancers. And CESC patients with USP28 alteration had poorer disease-free survival. In contrast, the UCEC and BLCA cases with altered USP28 had a better survival probability than the unaltered group (Figure 4D–4F). Moreover, mutations in MMR genes can disrupt the stability and integrity of the entire genome in normal cells [50], which also shows that USP28 plays a vital part in tumor growth and spread.

The process of DNA methylation, which is catalyzed by four DNMTs, can alter gene expression without changing the DNA sequence. This has emerged as a novel predictor for tumorigenesis [51]. We found that USP28 expression was highly associated with the DNMTs, vital in establishing and maintaining DNA methylation patterns [52]. Importantly, the promoter methylation level of USP28 was closely related to the USP28 expression, and the high methylation levels could result in decreased overall survival. Identifying aberrations in gene methylation patterns has emerged as a novel approach to predicting the development of cancers [53]. Therefore, the identification of aberrations in USP28 methylation patterns may provide a promising avenue for the development of molecular biomarkers for tumors.

Furthermore, we identified the USP28-related genes and signal pathways to reveal the mechanism of tumor progression. The GSEA data showed that USP28 was related to many immune-activated processes, including mitotic spindle, E2F targets, and G2M checkpoint pathways. Still, opposite findings were observed in different cancers. For example, these processes were most significantly enriched in high-USP28 cancer subgroups. Still, reversed results were found in CHOL, KICH, LAML, UCS, and UVM (Figure 6). The study by Oshi et al. found that the E2F pathway score is a predictive biomarker of response to neoadjuvant therapy in breast cancer [54]. They also discovered that the G2M checkpoint pathway alone is associated with drug response and survival among cell proliferation-related pathways in pancreatic cancer [55], which could support our findings somewhat.

Another significant finding of this study is that the expression of USP28 is significantly associated with immune infiltration in different types of cancers. Most cancers had a significant positive correlation between USP28 and neutrophil and NK T cell infiltration (Figure 7). Neutrophils have been found to support tumor progression by increasing tumor cell proliferation, promoting angiogenesis and stromal remodeling, and suppressing T cell-dependent antitumor response [56]. And neutrophil extracellular traps (NETs) were found to promote cancer cell growth and metastasis by trapping circulating cancer cells in distant inflamed organs [57]. The latest research has discovered that Cathepsin C promotes breast cancer lung metastasis by modulating neutrophil infiltration and neutrophil extracellular trap formation [58]. Therefore, our results indicate that USP28 could influence cancer development and prognosis by changing the tumor microenvironment.

Normally, the immune system is capable of identifying and eliminating cancerous cells. However, cancer cells can employ different survival and proliferation mechanisms, enabling them to evade detection and attack by the immune system. Fortunately, tumor immunotherapy has emerged as a promising approach to counteract the evasive tactics of cancer cells. This includes using monoclonal antibodies, immune checkpoint inhibitors, cancer vaccines, therapeutic antibodies, and cell-based therapies, which can help reinvigorate the body’s immune response and improve clinical outcomes for patients with various types of cancer [59]. Therefore, we further analyzed the correlation between immune checkpoint genes and USP28 expression. We found that USP28 expression is related to many immune regulator gene expressions in many cancers, including COAD, LIHC, PAAD, PRAD, and UVM (Figure 8A). And especially, USP28 was significantly correlated with NRP1, CD276, ADORA2A, and TNFSF15 in most cancers. Among them, CD276 [60], ADORA2A [61], and NRP1 [62] have achieved remarkable success in tumor immunotherapy, which suggested USP28 expression was linked with infiltration levels, which indicates that the potential relationship between USP28 and the above immune regulatory genes may be worthy of further investigation.

In addition to immune checkpoint regulators, TMB and MSI have emerged as novel biomarker candidates. Further, MSI was related to an increased risk of cancers [63]. TMB was considered to be related to more tumor neoantigens, which could facilitate immune recognition and promote an antitumor immune response, which also was a latent biomarker for predicting immune checkpoint blockade response [64]. In breast cancers, TMB could predict immune-mediated survival outcomes [65]. Similarly, MSI could be an important predictive factor for treatment outcomes of gastroesophageal adenocarcinoma [66]. This study illustrated that USP28 expression was significantly connected with TMB and MSI in most cancers (Figure 8). Thus, the specific mechanism of USP28 affecting immune checkpoint inhibitors, TMB, and MSI deserves further investigation. We also found the cohort with higher USP28 expression had a worse prognosis and resistance to anti-CTLA4 therapy. This study suggests USP28 was a powerful biomarker to predict response to immune checkpoint blockade therapy in pan-cancer. Therefore, we hypothesized that USP28 could be a powerful biomarker in predicting tumor immunotherapy effects. Our study evaluated the relationship between USP28 and clinical prognosis in cancer patients. A meaningful finding is that most cancers’ OS, DSS, DFI, and PFI analysis results were consistent (Figure 9). The study revealed that USP28 is a risk factor for 19 types of cancer patients and a protective factor for seven types of cancer. These findings demonstrate the significant role of USP28 in predicting the prognosis of cancer patients and suggest that it could serve as a powerful biomarker for predicting prognosis in cancer patients. Finally, the functional experiments confirmed that USP28 significantly promoted proliferation, invasion, and migration, which agrees with previous tumor studies [11, 67, 68]. These findings validate the accuracy and reliability of the pan-cancer analysis. Further molecular biological validation will be conducted in additional cancer types.

However, even though we incorporated some datasets to analyze the clinical significance of USP28 in pan-cancer analysis for the first time, this investigation still had several limitations. Initially, we obtained multiple datasets from different databases to perform our pan-cancer analysis, which may have introduced a degree of systematic bias. Moreover, USP28 expression is associated with cancer immunity and clinical survival prognosis. However, the specific signaling pathway of USP28 affecting clinical survival remains uncertain. Lastly, although we conducted cell experiments *in vitro* to explore the biological function of USP28, further biological experiments *in vivo* are still needed to validate our findings and accelerate clinical application. Nonetheless, our study provided a complete understanding of USP28, emphasizing the relationship between USP28 and tumor prognosis and tumor immunity across cancer types.

## CONCLUSIONS

In summary, our study has first confirmed that USP28 expression is a biomarker of the prognosis of cancers and can effectively predict immunotherapy response. In addition, the abnormal expression of USP28 was observed and more likely to correlate with clinical prognosis, protein phosphorylation, immune cell infiltration, immune checkpoints, tumor microenvironment, TMB, MSI, methylation, CNV, and MMR of multiple tumors. The experiments *in vitro* confirmed that USP28 could promote cell proliferation, migration, and invasion in the HCC cell lines. We concluded that USP28 could potentially be a prognostic marker and a novel target for tumor immunity in different cancers.

## Supplementary Material

Supplementary Figures

Supplementary Table 1

Supplementary Table 2

Supplementary Table 3
