# Supplementary material for: Comprehensive prognostic and immunological analysis of Ubiquitin Specific Peptidase 28 in pan-cancers and identification of its role in hepatocellular carcinoma cell lines
Source: Aging (Albany NY). 2023 Jul 13;15(13):6545–76. doi: 10.18632/aging.204869 (PMC10373984; doi:10.18632/aging.204869)
Supplement: Supplementary Figures [file aging-15-204869-s001.pdf]

SUPPLEMENTARY FIGURES

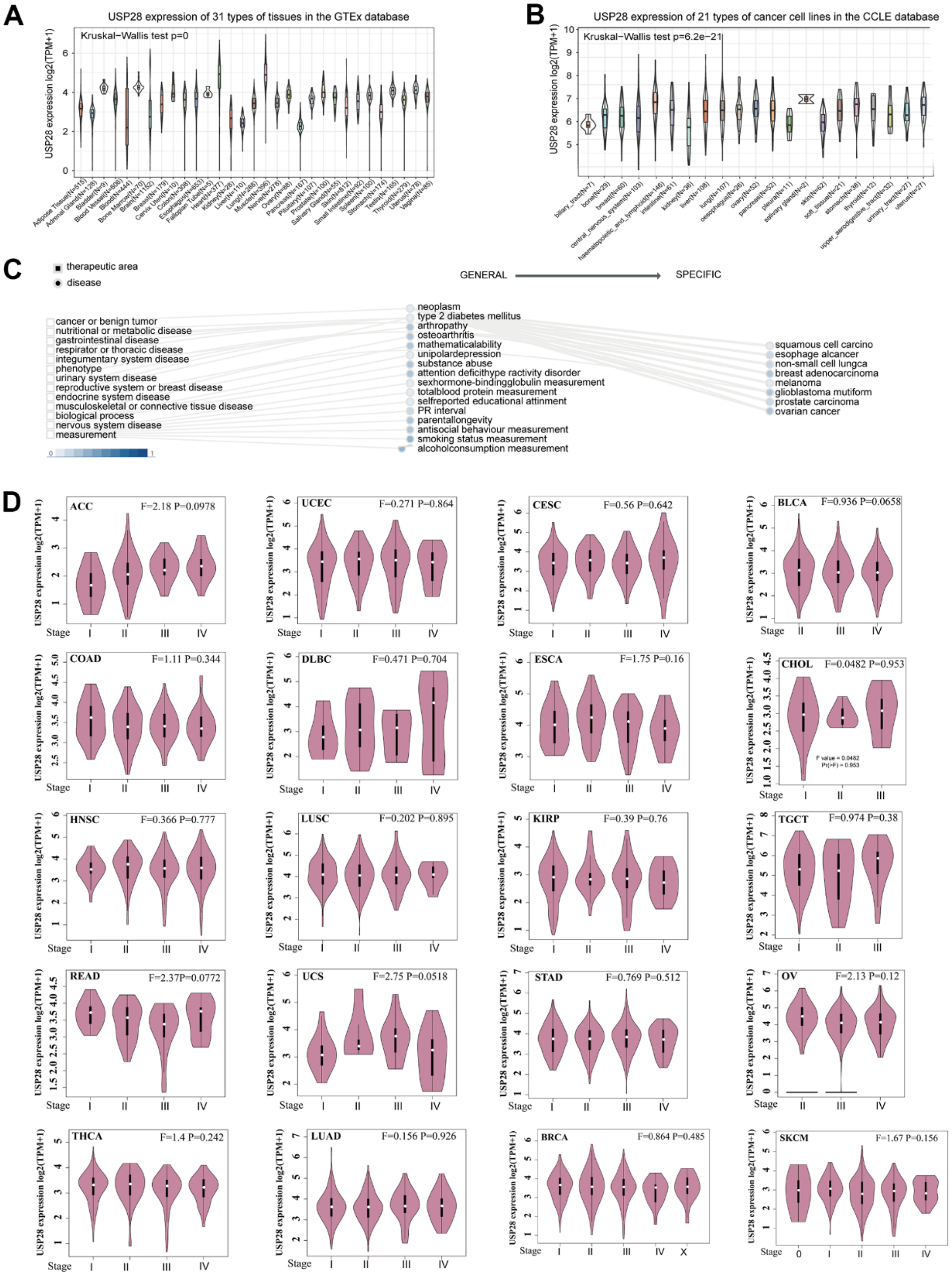

Supplementary Figure 1. Basic expression information of USP28. (**A**) USP28 expression levels in normal organs based on the GTEx dataset. (**B**) USP28 expression levels in diverse cancer cell lines from the data of the CCLE dataset. (**C**) The involvement of USP28 in diseases is based on the OPENTARGET platform. (**D**) The correlation between expression levels of USP28 and the main pathological stages (stages I, II, III, IV) in ACC, UCEC, CESC, BLCA, COAD, DLBC, ESCA, CHOL, HNSC, LUSC, KIRP, TGCT, READ, UCS, STAD, OV, THCA, LUAD, BRCA, and SKCM.

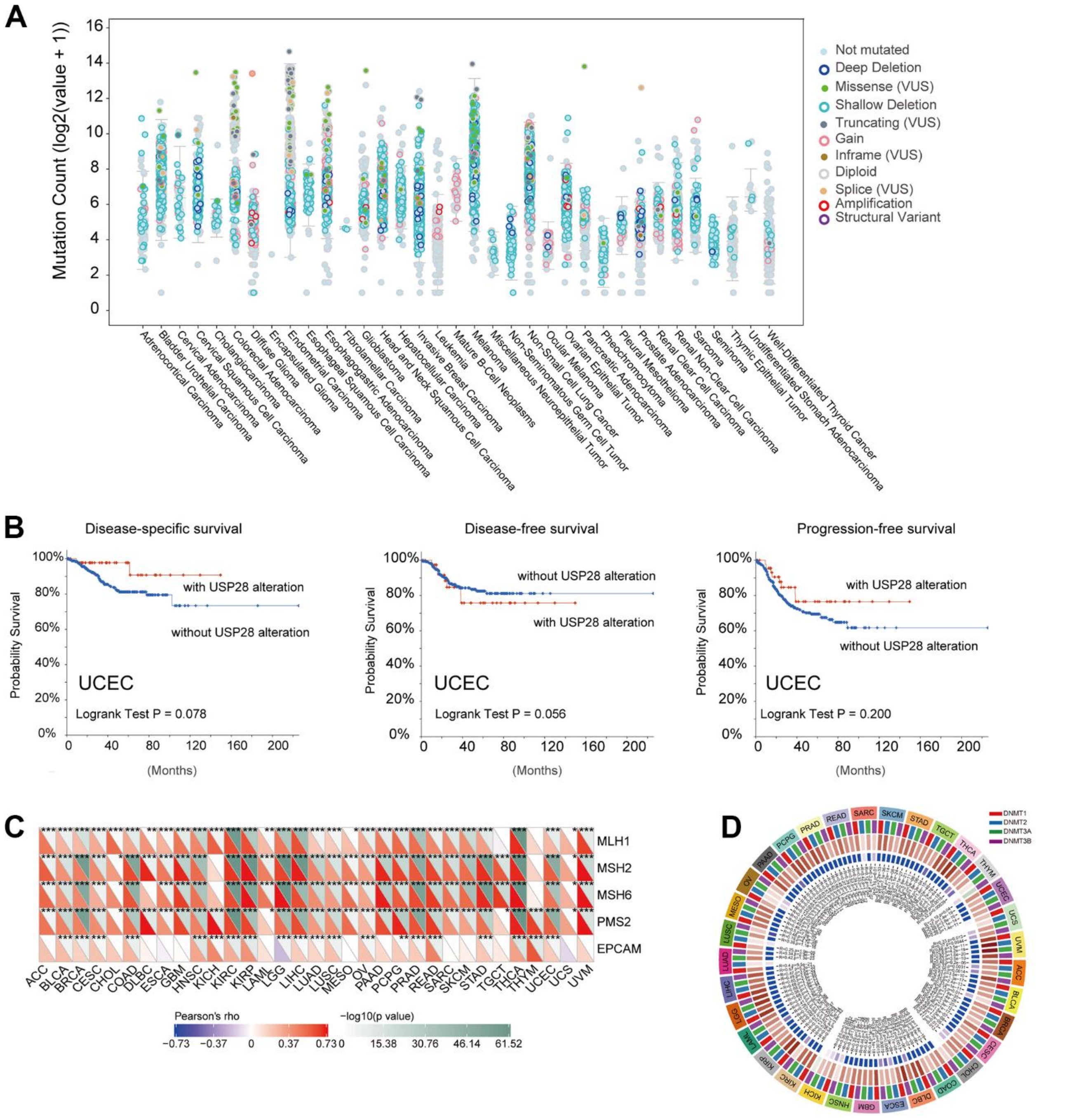

Supplementary Figure 2. The landscape of genetic alterations of USP28 in pan-cancer. (**A**) The entire mutation count of USP28 from the TCGA dataset is based on the cBioPortal tool. (**B**) The potential correlation between USP28 alteration and disease-specific, disease-free, progress-free survival in UCEC. (**C**) The correlations between USP28 expression and five MMR genes expression (MLH1, MSH2, MSH6, PMS2, and EPCAM) were described. (**D**) The associations between USP28 expression and four methylation transferases (DNMT1, DNMT2, DNMT3A, and DNMT3B) in different TCGA tumors were displayed. *$p < 0.05$; ** $p < 0.01$; *** $p < 0.001$.

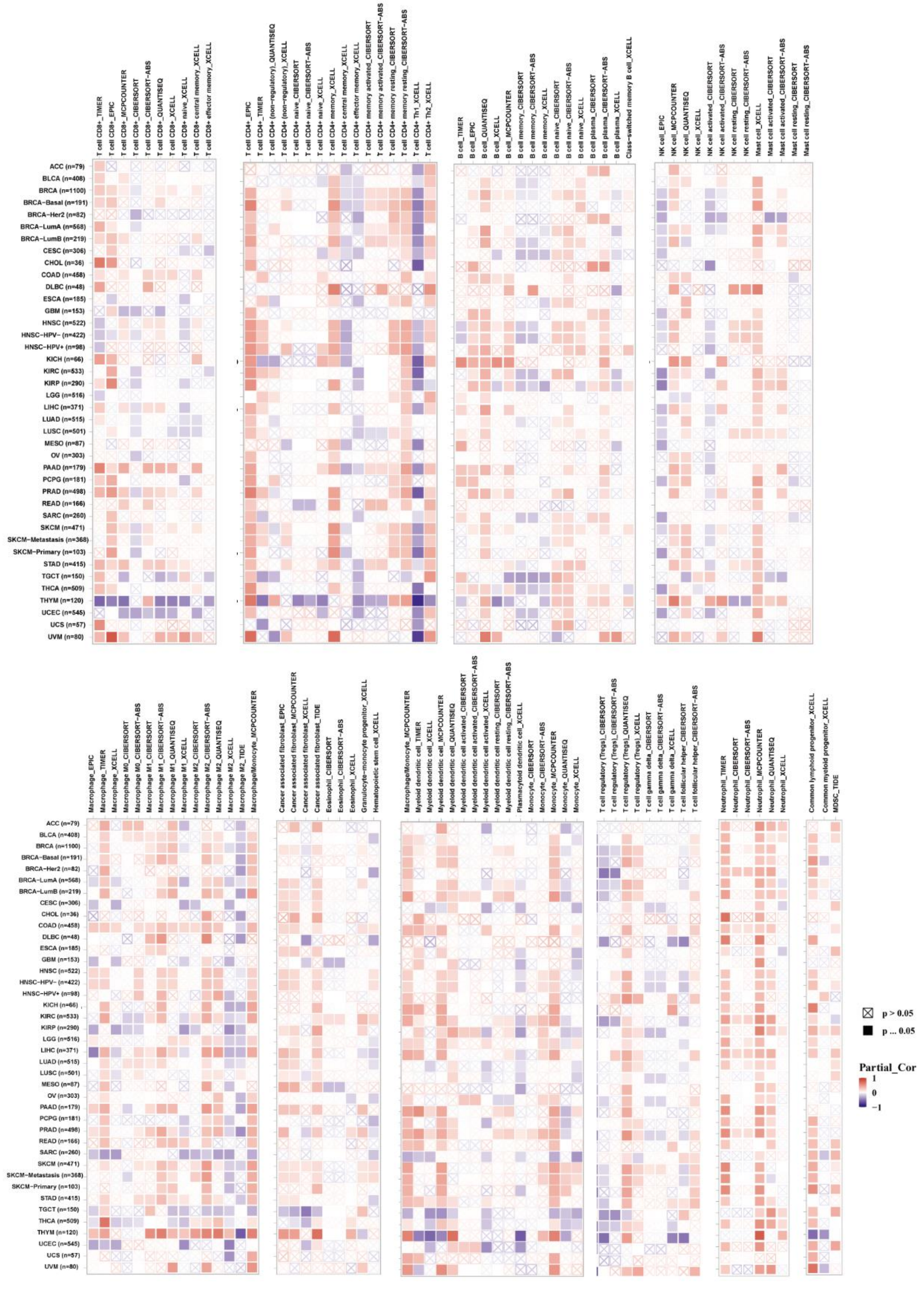

Supplementary Figure 3. Correlation between USP28 expression and immune cell infiltration in various pan-cancer types.

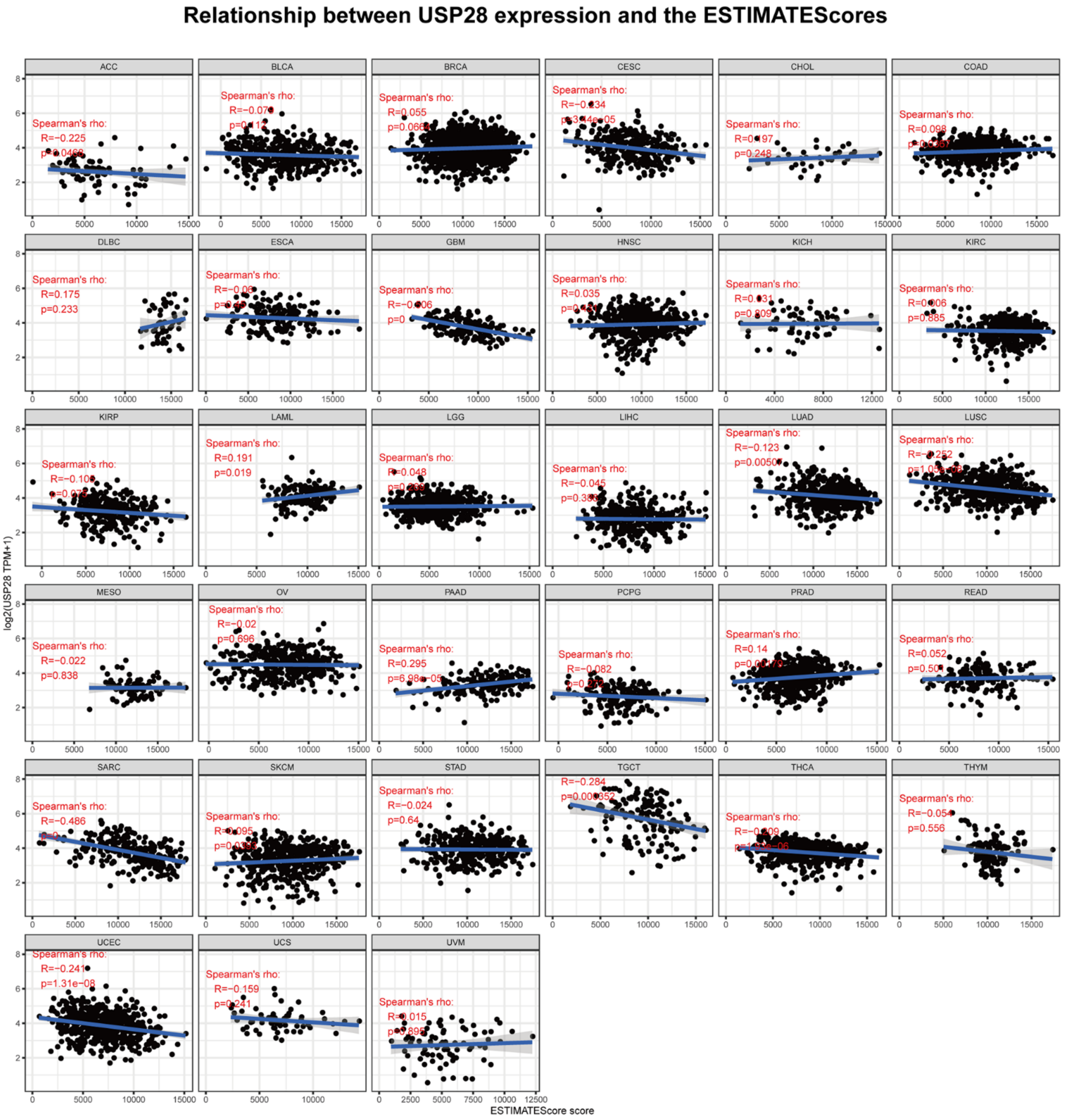

Supplementary Figure 4. Relationship between USP28 expression and EstimateScores in pan-cancer.

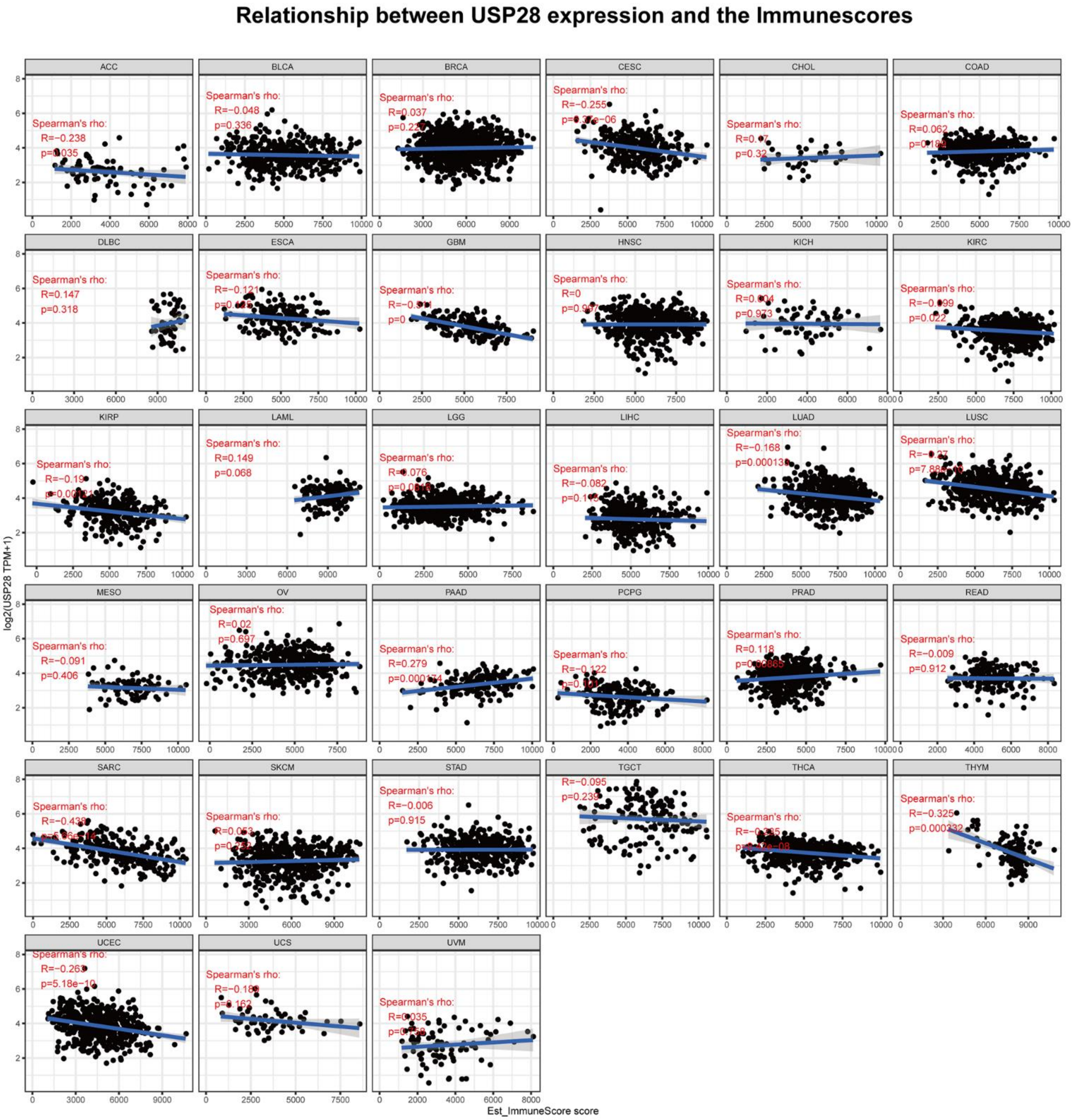

Supplementary Figure 5. Relationship between USP28 expression and ImmuneScores in pan-cancer.

Relationship between USP28 expression and the StromalScores.

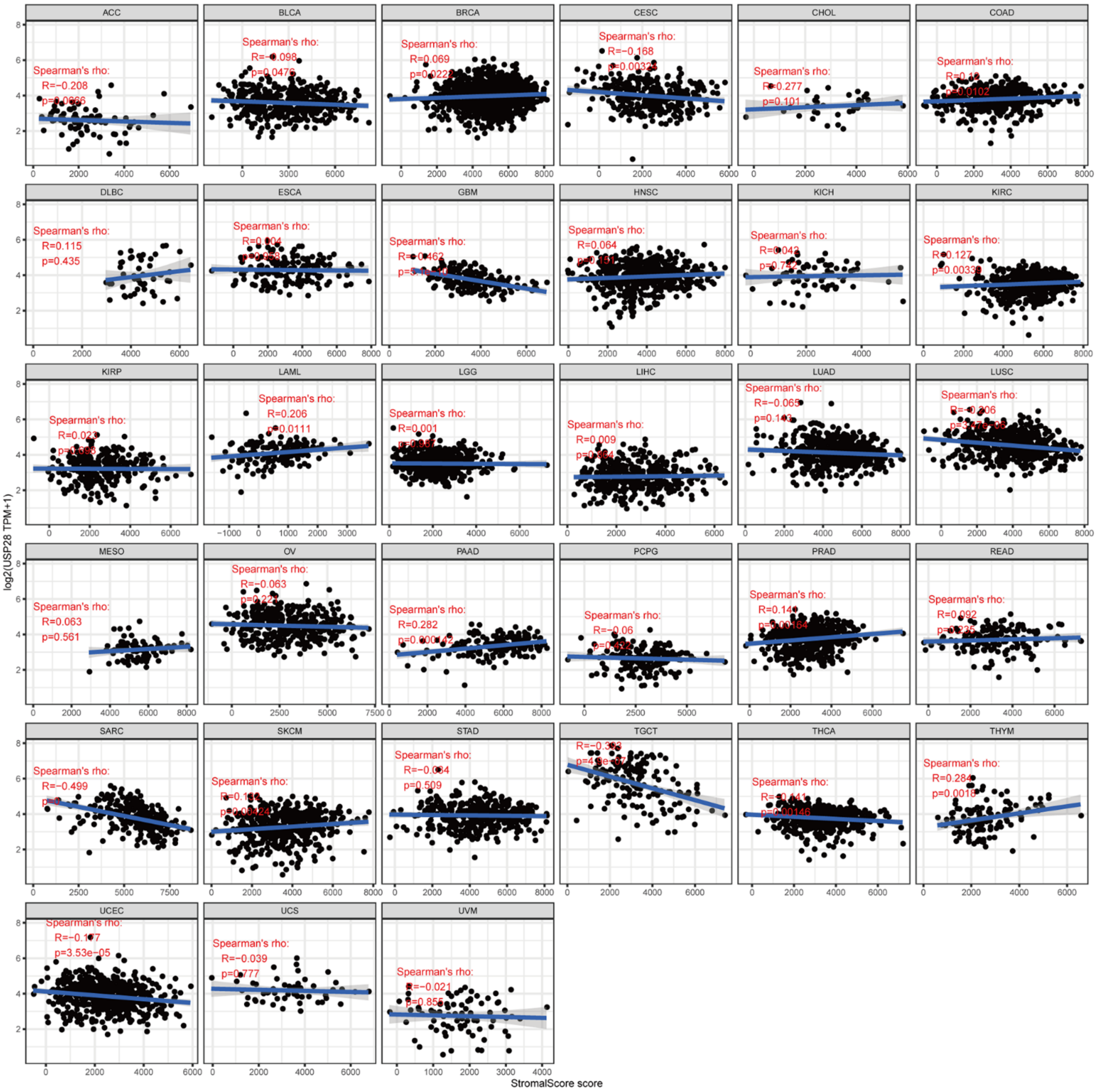

Supplementary Figure 6. Relationship between USP28 expression and StromalScores in pan-cancer.

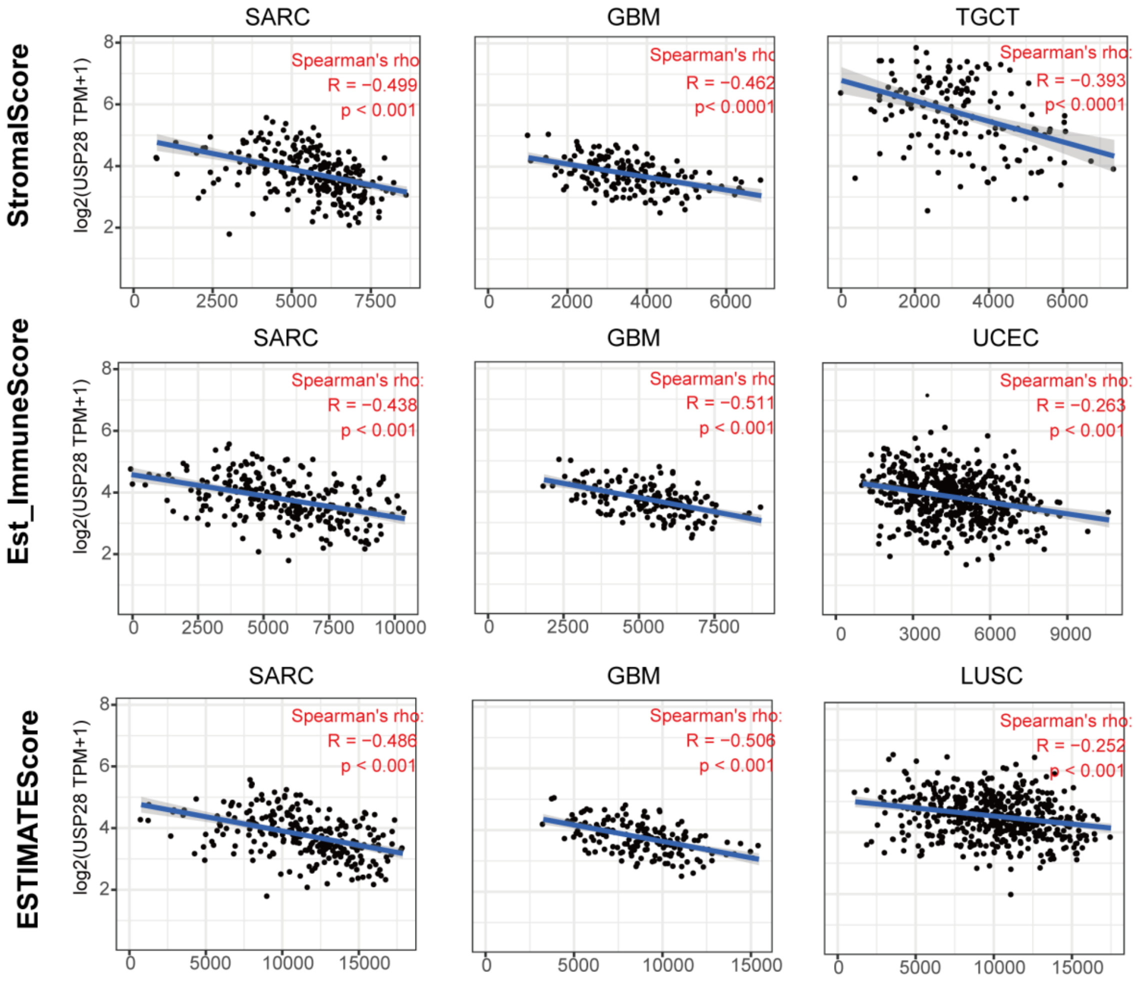

Supplementary Figure 7. Top three cancers related with StromalScore, ImmuneScore, and EstimateScore based on USP28 levels.

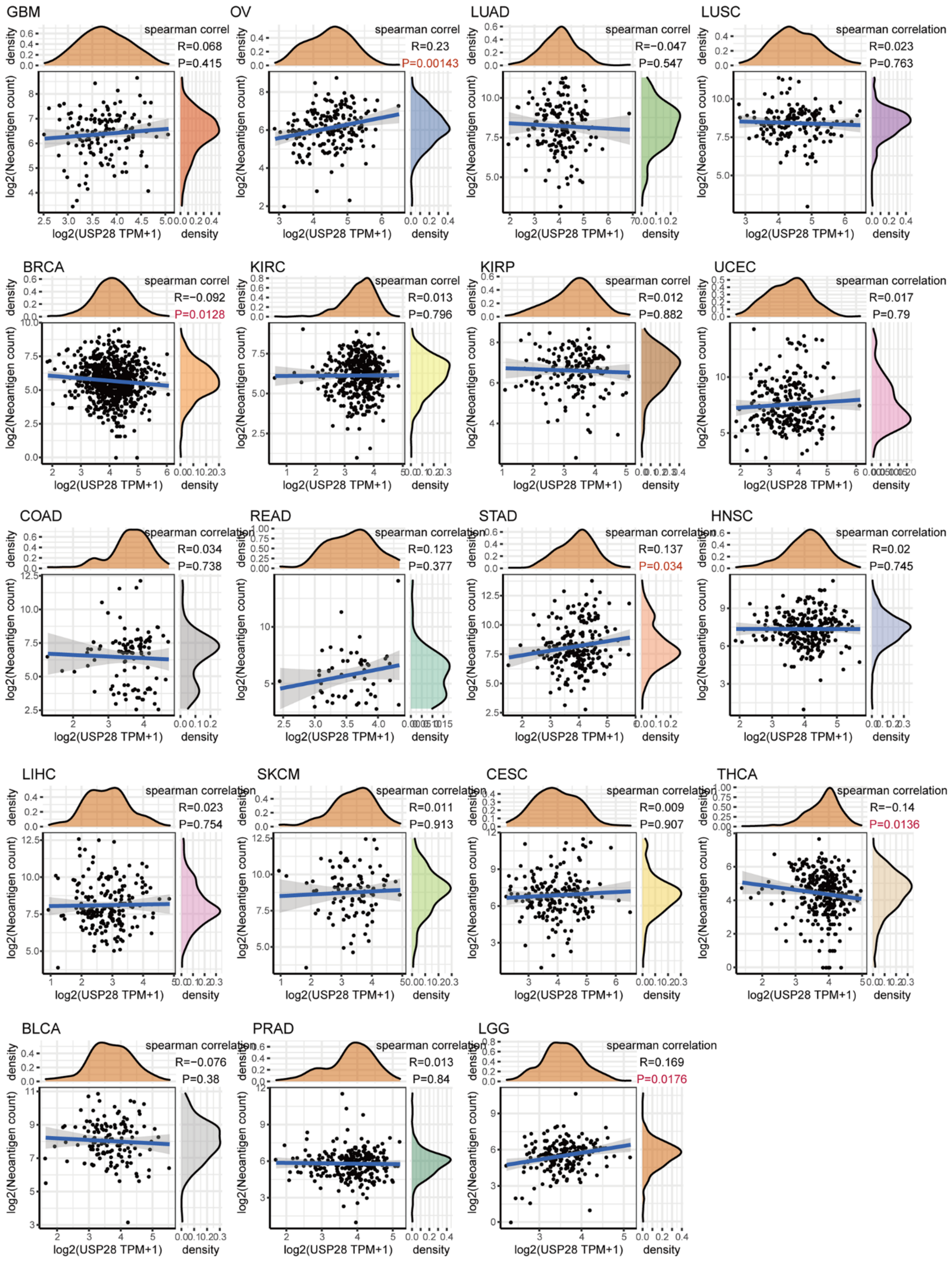

Supplementary Figure 8. Relationship between USP28 expression and neoantigens in pan-cancer.
