# Supplementary material for: Comprehensive prognostic and immunological analysis of Ubiquitin Specific Peptidase 28 in pan-cancers and identification of its role in hepatocellular carcinoma cell lines
Source: Aging (Albany NY). 2023 Jul 13;15(13):6545–76. doi: 10.18632/aging.204869 (PMC10373984; doi:10.18632/aging.204869)
Supplement: Supplementary Table 2 [file aging-15-204869-s003.docx]

Supplementary Table 2. Mutation spectrum of USP28 across tumor samples.

| **Sample ID** | **Cancer Type** | **Protein Change** | **Mutation Type** | **Copy #** | **Allele Freq (T)** |
| --- | --- | --- | --- | --- | --- |
| TCGA-DJ-A1QL-01 | Well-Differentiated Thyroid Cancer | R187* | Nonsense_ | Diploid | 0.31 |
| TCGA-ND-A4WC-01 | Endometrial Carcinoma | X46_splice | Splice_Site | Diploid | 0.14 |
| TCGA-OR-A5L2-01 | Adrenocortical Carcinoma | S718C | Missense_ | ShallowDel | 0.24 |
| TCGA-P5-A77X-01 | Diffuse Glioma | K209Efs*21 | Frame_Shift_Del | Diploid | 0.31 |
| TCGA-DU-6392-01 | Diffuse Glioma | X954_splice | Splice_Site | Gain | 0.21 |
| TCGA-DU-6392-01 | Diffuse Glioma | V944A | Missense_ | Gain | 0.07 |
| TCGA-DU-6392-01 | Diffuse Glioma | F370S | Missense_ | Gain | 0.44 |
| TCGA-DU-7294-01 | Diffuse Glioma | S347* | Nonsense_ | Diploid | 0.07 |
| TCGA-04-1651-01 | Ovarian Epithelial Tumor | W888* | Nonsense_ | Diploid | 0.36 |
| TCGA-29-1761-01 | Ovarian Epithelial Tumor | R428T | Missense_ | ShallowDel | 0.15 |
| TCGA-28-2509-01 | Glioblastoma | A1002T | Missense_ | ShallowDel | 0.31 |
| TCGA-27-1832-01 | Glioblastoma | S453G | Missense_ | Diploid | 0.33 |
| TCGA-06-5416-01 | Glioblastoma | A870V | Missense_ | Diploid | 0.44 |
| TCGA-15-1446-01 | Glioblastoma | T298N | Missense_ | Diploid | 0.04 |
| TCGA-DX-A7EL-01 | Sarcoma | K138T | Missense_ | Diploid | 0.31 |
| TCGA-77-6844-01 | Non-Small Cell Lung Cancer | G599E | Missense_ | Diploid | 0.34 |
| TCGA-60-2719-01 | Non-Small Cell Lung Cancer | L825F | Missense_ | Gain | 0.36 |
| TCGA-66-2759-01 | Non-Small Cell Lung Cancer | E693Q | Missense_ | Gain | 0.16 |
| TCGA-46-6025-01 | Non-Small Cell Lung Cancer | D632Efs*4 | Frame_Shift_Del | ShallowDel | 0.16 |
| TCGA-96-8169-01 | Non-Small Cell Lung Cancer | E201* | Nonsense_ | ShallowDel | 0.28 |
| TCGA-O2-A52N-01 | Non-Small Cell Lung Cancer | A739S | Missense_ | ShallowDel | 0.14 |
| TCGA-XC-AA0X-01 | Non-Small Cell Lung Cancer | R155G | Missense_ | Diploid | 0.1 |
| TCGA-4Z-AA7O-01 | Bladder Urothelial Carcinoma | G120Efs*45 | Frame_Shift_Del | ShallowDel | 0.41 |
| TCGA-DK-AA71-01 | Bladder Urothelial Carcinoma | X887_splice | Splice_Site | Diploid | 0.37 |
| TCGA-DK-AA74-01 | Bladder Urothelial Carcinoma | X887_splice | Splice_Site | Diploid | 0.07 |
| TCGA-FD-A6TF-01 | Bladder Urothelial Carcinoma | C411F | Missense_ | Diploid | 0.13 |
| TCGA-GV-A3QI-01 | Bladder Urothelial Carcinoma | K420Nfs*30 | Frame_Shift_Del | Diploid | 0.36 |
| TCGA-KQ-A41N-01 | Bladder Urothelial Carcinoma | H970N | Missense_ | Diploid | 0.13 |
| TCGA-KQ-A41N-01 | Bladder Urothelial Carcinoma | H592N | Missense_ | Diploid | 0.13 |
| TCGA-SY-A9G5-01 | Bladder Urothelial Carcinoma | D618N | Missense_ | ShallowDel | 0.1 |
| TCGA-UY-A78M-01 | Bladder Urothelial Carcinoma | E693* | Nonsense_ | Gain | 0.22 |
| TCGA-XF-A8HD-01 | Bladder Urothelial Carcinoma | E268Q | Missense_ | Diploid | 0.4 |
| TCGA-ZF-A9R4-01 | Bladder Urothelial Carcinoma | R156T | Missense_ | ShallowDel | 0.4 |
| TCGA-GV-A3JZ-01 | Bladder Urothelial Carcinoma | R406Q | Missense_ | DeepDel | 0.76 |
| TCGA-BT-A0YX-01 | Bladder Urothelial Carcinoma | Q390* | Nonsense_ | Diploid | 0.16 |
| TCGA-GV-A3JX-01 | Bladder Urothelial Carcinoma | E965K | Missense_ | Diploid | 0.37 |
| TCGA-2F-A9KO-01 | Bladder Urothelial Carcinoma | Q496* | Nonsense_ | ShallowDel | 0.33 |
| TCGA-XF-AAN2-01 | Bladder Urothelial Carcinoma | X178_splice | Splice_Region | ShallowDel | 0.57 |
| TCGA-DD-AAEI-01 | Hepatocellular Carcinoma | W613R | Missense_ | Diploid | 0.26 |
| TCGA-EJ-5518-01 | Prostate Adenocarcinoma | S228* | Nonsense_ | ShallowDel | 0.04 |
| TCGA-J4-AAU2-01 | Prostate Adenocarcinoma | W145* | Nonsense_ | Diploid | 0.35 |
| TCGA-XK-AAIW-01 | Prostate Adenocarcinoma | A245T | Missense_ | Diploid | 0.37 |
| TCGA-XK-AAIW-01 | Prostate Adenocarcinoma | R824Q | Missense_ | Diploid | 0.39 |
| TCGA-XK-AAIW-01 | Prostate Adenocarcinoma | X582_splice | Splice_Site | Diploid | 0.27 |
| TCGA-J4-8200-01 | Prostate Adenocarcinoma | E630_R631insF | In_Frame_Ins | Diploid | 0.09 |
| TCGA-B8-5551-01 | Renal Clear Cell Carcinoma | N407* | Frame_Shift_Del | Diploid | 0.18 |
| TCGA-B0-5693-01 | Renal Clear Cell Carcinoma | V629G | Missense_ | Diploid | 0.05 |
| TCGA-W2-A7HD-01 | Pheochromocytoma | D338G | Missense_ | Diploid | 0.58 |
| TCGA-AP-A0LM-01 | Endometrial Carcinoma | R732C | Missense_ | Diploid | 0.1 |
| TCGA-EO-A3B0-01 | Endometrial Carcinoma | R413Q | Missense_ | Diploid | 0.35 |
| TCGA-AX-A3G9-01 | Endometrial Carcinoma | R585C | Missense_ | Diploid | 0.4 |
| TCGA-EY-A1GI-01 | Endometrial Carcinoma | H592N | Missense_ | Diploid | 0.39 |
| TCGA-BS-A0VI-01 | Endometrial Carcinoma | P235L | Missense_ | Diploid | 0.35 |
| TCGA-AP-A1DK-01 | Endometrial Carcinoma | A270T | Missense_ | Diploid | 0.38 |
| TCGA-AX-A2HG-01 | Endometrial Carcinoma | A105V | Missense_ | Diploid | 0.47 |
| TCGA-AJ-A3EL-01 | Endometrial Carcinoma | R204* | Nonsense_ | Diploid | 0.22 |
| TCGA-AX-A0J1-01 | Endometrial Carcinoma | R204* | Nonsense_ | Diploid | 0.35 |
| TCGA-EO-A22R-01 | Endometrial Carcinoma | R204* | Nonsense_ | Diploid | 0.31 |
| TCGA-D1-A16X-01 | Endometrial Carcinoma | R141C | Missense_ | Diploid | 0.04 |
| TCGA-A5-A2K5-01 | Endometrial Carcinoma | R406* | Nonsense_ | Diploid | 0.23 |
| TCGA-AJ-A3BH-01 | Endometrial Carcinoma | R247* | Nonsense_ | Diploid | 0.29 |
| TCGA-A5-A2K5-01 | Endometrial Carcinoma | R413* | Nonsense_ | Diploid | 0.61 |
| TCGA-AX-A2HC-01 | Endometrial Carcinoma | R413* | Nonsense_ | Diploid | 0.41 |
| TCGA-QS-A5YQ-01 | Endometrial Carcinoma | R413* | Nonsense_ | Diploid | 0.34 |
| TCGA-A5-A0GW-01 | Endometrial Carcinoma | V940Sfs*5 | Frame_Shift_Del | Diploid | 0.22 |
| TCGA-EO-A22X-01 | Endometrial Carcinoma | R369I | Missense_ | Diploid | 0.44 |
| TCGA-E6-A1LX-01 | Endometrial Carcinoma | R1050Q | Missense_ | Diploid | 0.12 |
| TCGA-BS-A0UV-01 | Endometrial Carcinoma | R302C | Missense_ | Diploid | 0.4 |
| TCGA-D1-A17Q-01 | Endometrial Carcinoma | R302C | Missense_ | Diploid | 0.41 |
| TCGA-D1-A167-01 | Endometrial Carcinoma | R840G | Missense_ | Diploid | 0.13 |
| TCGA-AP-A051-01 | Endometrial Carcinoma | R187* | Nonsense_ | Diploid | 0.31 |
| TCGA-B5-A1MW-01 | Endometrial Carcinoma | A764V | Missense_ | Diploid | 0.43 |
| TCGA-A5-A0G2-01 | Endometrial Carcinoma | R324H | Missense_ | Diploid | 0.23 |
| TCGA-B5-A11G-01 | Endometrial Carcinoma | E805D | Missense_ | Diploid | 0.35 |
| TCGA-AP-A051-01 | Endometrial Carcinoma | R844* | Nonsense_ | Diploid | 0.13 |
| TCGA-AP-A059-01 | Endometrial Carcinoma | R844* | Nonsense_ | Diploid | 0.21 |
| TCGA-AJ-A5DW-01 | Endometrial Carcinoma | R210I | Missense_ | Diploid | 0.4 |
| TCGA-B5-A0JY-01 | Endometrial Carcinoma | R210I | Missense_ | Diploid | 0.32 |
| TCGA-D1-A17Q-01 | Endometrial Carcinoma | R210I | Missense_ | Diploid | 0.27 |
| TCGA-D1-A17Q-01 | Endometrial Carcinoma | V582A | Missense_ | Diploid | 0.27 |
| TCGA-A5-A0G2-01 | Endometrial Carcinoma | C1011* | Nonsense_ | Diploid | 0.23 |
| TCGA-AP-A059-01 | Endometrial Carcinoma | C1011* | Nonsense_ | Diploid | 0.16 |
| TCGA-AX-A1CE-01 | Endometrial Carcinoma | C1011* | Nonsense_ | Diploid | 0.38 |
| TCGA-D1-A101-01 | Endometrial Carcinoma | X179_splice | Splice_Site | Diploid | 0.12 |
| TCGA-BS-A0UF-01 | Endometrial Carcinoma | D891E | Missense_ | Diploid | 0.34 |
| TCGA-D1-A103-01 | Endometrial Carcinoma | K959R | Missense_ | Diploid | 0.1 |
| TCGA-A5-A0G2-01 | Endometrial Carcinoma | G975D | Missense_ | Diploid | 0.42 |
| TCGA-A5-A0G2-01 | Endometrial Carcinoma | E694K | Missense_ | Diploid | 0.24 |
| TCGA-A5-A0G2-01 | Endometrial Carcinoma | N282S | Missense_ | Diploid | 0.2 |
| TCGA-A5-A0G2-01 | Endometrial Carcinoma | M126I | Missense_ | Diploid | 0.25 |
| TCGA-A5-A1OF-01 | Endometrial Carcinoma | R125M | Missense_ | Diploid | 0.42 |
| TCGA-AJ-A3BG-01 | Endometrial Carcinoma | L426S | Missense_ | Diploid | 0.54 |
| TCGA-AJ-A3BH-01 | Endometrial Carcinoma | L471S | Missense_ | Diploid | 0.3 |
| TCGA-AJ-A3EK-01 | Endometrial Carcinoma | P653S | Missense_ | Diploid | 0.47 |
| TCGA-AP-A1DK-01 | Endometrial Carcinoma | R786L | Missense_ | Diploid | 0.29 |
| TCGA-AP-A1E0-01 | Endometrial Carcinoma | F372C | Missense_ | Diploid | 0.33 |
| TCGA-AX-A1CE-01 | Endometrial Carcinoma | A333S | Missense_ | Diploid | 0.41 |
| TCGA-B5-A0JV-01 | Endometrial Carcinoma | N166Kfs*9 | Frame_Shift_Ins | Diploid | 0.33 |
| TCGA-B5-A1MX-01 | Endometrial Carcinoma | L473F | Missense_ | Diploid | 0.21 |
| TCGA-B5-A3FC-01 | Endometrial Carcinoma | R938Q | Missense_ | Diploid | 0.43 |
| TCGA-B5-A3FC-01 | Endometrial Carcinoma | P510S | Missense_ | Diploid | 0.32 |
| TCGA-D1-A17Q-01 | Endometrial Carcinoma | D122Y | Missense_ | Diploid | 0.07 |
| TCGA-DF-A2KU-01 | Endometrial Carcinoma | D891Y | Missense_ | Diploid | 0.4 |
| TCGA-DF-A2KU-01 | Endometrial Carcinoma | S714P | Missense_ | Diploid | 0.38 |
| TCGA-DF-A2KZ-01 | Endometrial Carcinoma | P161S | Missense_ | Diploid | 0.34 |
| TCGA-DI-A1BU-01 | Endometrial Carcinoma | Q424R | Missense_ | Diploid | 0.28 |
| TCGA-DI-A1BU-01 | Endometrial Carcinoma | X254_splice | Splice_Site | Diploid | 0.48 |
| TCGA-DI-A1NO-01 | Endometrial Carcinoma | X768_splice | Splice_Region | Diploid | 0.32 |
| TCGA-E6-A1LX-01 | Endometrial Carcinoma | K420N | Missense_ | Diploid | 0.16 |
| TCGA-EO-A22R-01 | Endometrial Carcinoma | D994G | Missense_ | Diploid | 0.4 |
| TCGA-EO-A22X-01 | Endometrial Carcinoma | S927N | Missense_ | Diploid | 0.43 |
| TCGA-EO-A22X-01 | Endometrial Carcinoma | I741S | Missense_ | Diploid | 0.32 |
| TCGA-EO-A22X-01 | Endometrial Carcinoma | D507N | Missense_ | Diploid | 0.39 |
| TCGA-EO-A3AY-01 | Endometrial Carcinoma | F292I | Missense_ | Diploid | 0.39 |
| TCGA-EO-A3B0-01 | Endometrial Carcinoma | E1004* | Nonsense_ | Diploid | 0.41 |
| TCGA-EY-A548-01 | Endometrial Carcinoma | Q527Sfs*12 | Frame_Shift_Del | Diploid | 0.15 |
| TCGA-17-Z026-01 | Non-Small Cell Lung Cancer | Q682* | Nonsense_ | NA | 0.08 |
| TCGA-05-4396-01 | Non-Small Cell Lung Cancer | Q178* | Nonsense_ | Diploid | 0.35 |
| TCGA-55-6985-01 | Non-Small Cell Lung Cancer | R121I | Missense_ | Diploid | 0.08 |
| TCGA-44-4112-01 | Non-Small Cell Lung Cancer | C329* | Nonsense_ | ShallowDel | 0.47 |
| TCGA-78-7155-01 | Non-Small Cell Lung Cancer | P1047Q | Missense_ | Gain | 0.26 |
| TCGA-78-7536-01 | Non-Small Cell Lung Cancer | G935V | Missense_ | Diploid | 0.21 |
| TCGA-55-7283-01 | Non-Small Cell Lung Cancer | A764V | Missense_ | ShallowDel | 0.21 |
| TCGA-38-4625-01 | Non-Small Cell Lung Cancer | D852Y | Missense_ | ShallowDel | 0.22 |
| TCGA-55-6969-01 | Non-Small Cell Lung Cancer | G599V | Missense_ | Diploid | 0.15 |
| TCGA-55-8205-01 | Non-Small Cell Lung Cancer | Q197* | Nonsense_ | Diploid | 0.26 |
| TCGA-62-8395-01 | Non-Small Cell Lung Cancer | S475* | Nonsense_ | ShallowDel | 0.11 |
| TCGA-62-A46O-01 | Non-Small Cell Lung Cancer | D999Y | Missense_ | Diploid | 0.5 |
| TCGA-05-4244-01 | Non-Small Cell Lung Cancer | X304_splice | Splice_Region | DeepDel | 0.24 |
| TCGA-17-Z045-01 | Non-Small Cell Lung Cancer | G797V | Missense_ | NA | 0.52 |
| TCGA-JY-A6FG-01 | Esophageal Squamous Cell Carcinoma | T312A | Missense_ | Diploid | 0.26 |
| TCGA-LN-A49M-01 | Esophageal Squamous Cell Carcinoma | E974K | Missense_ | ShallowDel | 0.59 |
| TCGA-R6-A6L6-01 | Esophagogastric Adenocarcinoma | W626S | Missense_ | Gain | 0.2 |
| TCGA-FS-A4FC-06 | Melanoma | S919F | Missense_ | Diploid | 0.21 |
| TCGA-EB-A5SE-01 | Melanoma | P389S | Missense_ | NA | 0.41 |
| TCGA-W3-A825-06 | Melanoma | R585C | Missense_ | Diploid | 0.49 |
| TCGA-FW-A3R5-06 | Melanoma | R406Q | Missense_ | Diploid | 0.2 |
| TCGA-D3-A2JF-06 | Melanoma | R324C | Missense_ | ShallowDel | 0.65 |
| TCGA-EE-A2MP-06 | Melanoma | V940Gfs*26 | Frame_Shift_Ins | ShallowDel | 0.25 |
| TCGA-D3-A3ML-06 | Melanoma | P317S | Missense_ | ShallowDel | 0.87 |
| TCGA-EE-A2MI-06 | Melanoma | P317S | Missense_ | Diploid | 0.4 |
| TCGA-FS-A1ZA-06 | Melanoma | F365S | Missense_ | ShallowDel | 0.62 |
| TCGA-FW-A5DY-06 | Melanoma | P343S | Missense_ | Diploid | 0.07 |
| TCGA-EE-A2MS-06 | Melanoma | N124D | Missense_ | ShallowDel | 0.75 |
| TCGA-GF-A3OT-06 | Melanoma | N854K | Missense_ | Diploid | 0.22 |
| TCGA-EE-A3J7-06 | Melanoma | L180F | Missense_ | ShallowDel | 0.73 |
| TCGA-BF-A3DM-01 | Melanoma | S943F | Missense_ | NA | 0.43 |
| TCGA-FR-A726-01 | Melanoma | Q915* | Nonsense_ | NA | 0.5 |
| TCGA-EE-A3JD-06 | Melanoma | L953F | Missense_ | Diploid | 0.09 |
| TCGA-EE-A2MD-06 | Melanoma | S996F | Missense_ | ShallowDel | 0.45 |
| TCGA-FW-A3R5-06 | Melanoma | Q666* | Nonsense_ | Diploid | 0.28 |
| TCGA-FW-A3R5-06 | Melanoma | M706I | Missense_ | Diploid | 0.29 |
| TCGA-ER-A42L-06 | Melanoma | P838L | Missense_ | ShallowDel | 0.11 |
| TCGA-D3-A2J6-06 | Melanoma | E916D | Missense_ | ShallowDel | 0.1 |
| TCGA-D3-A2J7-06 | Melanoma | R786S | Missense_ | ShallowDel | 0.13 |
| TCGA-D3-A2JL-06 | Melanoma | R324S | Missense_ | ShallowDel | 0.09 |
| TCGA-D3-A8GM-06 | Melanoma | S819A | Missense_ | ShallowDel | 0.37 |
| TCGA-EE-A181-06 | Melanoma | G793V | Missense_ | Diploid | 0.11 |
| TCGA-EE-A183-06 | Melanoma | H1009N | Missense_ | Diploid | 0.1 |
| TCGA-EE-A29E-06 | Melanoma | L426F | Missense_ | Diploid | 0.09 |
| TCGA-EE-A2MD-06 | Melanoma | Q319K | Missense_ | ShallowDel | 0.12 |
| TCGA-ER-A19C-06 | Melanoma | Q557K | Missense_ | ShallowDel | 0.09 |
| TCGA-FS-A1ZG-06 | Melanoma | M522I | Missense_ | Diploid | 0.09 |
| TCGA-WE-A8K5-06 | Melanoma | S508F | Missense_ | ShallowDel | 0.26 |
| TCGA-WE-A8ZX-06 | Melanoma | Q182* | Nonsense_ | Gain | 0.41 |
| TCGA-BR-7851-01 | Esophagogastric Adenocarcinoma | P235L | Missense_ | Diploid | 0.23 |
| TCGA-D7-A6EY-01 | Esophagogastric Adenocarcinoma | R141C | Missense_ | Diploid | 0.2 |
| TCGA-F1-6177-01 | Esophagogastric Adenocarcinoma | V940Sfs*5 | Frame_Shift_Del | Diploid | 0.26 |
| TCGA-VQ-A8P2-01 | Esophagogastric Adenocarcinoma | R1050Q | Missense_ | Diploid | 0.34 |
| TCGA-VQ-A8P2-01 | Esophagogastric Adenocarcinoma | R141H | Missense_ | Diploid | 0.08 |
| TCGA-F1-A448-01 | Esophagogastric Adenocarcinoma | R136H | Missense_ | Diploid | 0.16 |
| TCGA-BR-6706-01 | Esophagogastric Adenocarcinoma | R896Q | Missense_ | Diploid | 0.13 |
| TCGA-CD-A4MG-01 | Esophagogastric Adenocarcinoma | R896Q | Missense_ | Diploid | 0.13 |
| TCGA-D7-A4YT-01 | Esophagogastric Adenocarcinoma | E201* | Nonsense_ | ShallowDel | 0.49 |
| TCGA-HU-A4GQ-01 | Esophagogastric Adenocarcinoma | G761S | Missense_ | Diploid | 0.25 |
| TCGA-BR-6452-01 | Esophagogastric Adenocarcinoma | X304_splice | Splice_Site | Diploid | 0.1 |
| TCGA-CG-5721-01 | Esophagogastric Adenocarcinoma | L982P | Missense_ | Diploid | 0.17 |
| TCGA-CG-5721-01 | Esophagogastric Adenocarcinoma | S401C | Missense_ | Diploid | 0.18 |
| TCGA-D7-A6EV-01 | Esophagogastric Adenocarcinoma | R519W | Missense_ | Gain | 0.21 |
| TCGA-BR-8680-01 | Esophagogastric Adenocarcinoma | S1053Y | Missense_ | Diploid | 0.33 |
| TCGA-B7-5816-01 | Esophagogastric Adenocarcinoma | A333Pfs*16 | Frame_Shift_Del | Diploid | 0.09 |
| TCGA-IN-AB1V-01 | Esophagogastric Adenocarcinoma | I750M | Missense_ | Diploid | 0.2 |
| TCGA-BR-6458-01 | Esophagogastric Adenocarcinoma | P379A | Missense_ | Diploid | 0.22 |
| TCGA-BR-8081-01 | Esophagogastric Adenocarcinoma | A333V | Missense_ | Diploid | 0.19 |
| TCGA-VQ-A91K-01 | Esophagogastric Adenocarcinoma | R937C | Missense_ | Diploid | 0.41 |
| TCGA-W5-AA2U-01 | Cholangiocarcinoma | K913N | Missense_ | ShallowDel | 0.54 |
| TCGA-AR-A2LE-01 | Invasive Breast Carcinoma | S248L | Missense_ | Diploid | 0.39 |
| TCGA-BH-A0HP-01 | Invasive Breast Carcinoma | Q53* | Nonsense_ | ShallowDel | 0.33 |
| TCGA-E9-A244-01 | Invasive Breast Carcinoma | P235L | Missense_ | Gain | 0.55 |
| TCGA-AC-A23H-01 | Invasive Breast Carcinoma | E311* | Nonsense_ | Diploid | 0.24 |
| TCGA-AC-A23H-01 | Invasive Breast Carcinoma | D255H | Missense_ | Diploid | 0.33 |
| TCGA-BH-A18G-01 | Invasive Breast Carcinoma | T845A | Missense_ | Diploid | 0.04 |
| TCGA-C8-A8HQ-01 | Invasive Breast Carcinoma | Q581H | Missense_ | ShallowDel | 0.36 |
| TCGA-AN-A0AK-01 | Invasive Breast Carcinoma | E768D | Missense_ | ShallowDel | 0.04 |
| TCGA-EW-A2FV-01 | Invasive Breast Carcinoma | S344Pfs*5 | Frame_Shift_Del | Diploid | 0.09 |
| TCGA-EW-A6SB-01 | Invasive Breast Carcinoma | I1003T | Missense_ | Gain | 0.1 |
| TCGA-IB-7651-01 | Pancreatic Adenocarcinoma | C644Y | Missense_ | Diploid | 0.13 |
| TCGA-IB-7651-01 | Pancreatic Adenocarcinoma | H477Y | Missense_ | Diploid | 0.2 |
| TCGA-IB-7651-01 | Pancreatic Adenocarcinoma | D269G | Missense_ | Diploid | 0.06 |
| TCGA-HV-A5A6-01 | Pancreatic Adenocarcinoma | X860_splice | Splice_Site | Diploid | 0.27 |
| TCGA-2L-AAQM-01 | Pancreatic Adenocarcinoma | G761S | Missense_ | ShallowDel | 0.82 |
| TCGA-AA-3984-01 | Colorectal Adenocarcinoma | R732C | Missense_ | Diploid | 0.1 |
| TCGA-A6-5661-01 | Colorectal Adenocarcinoma | Q581H | Missense_ | Diploid | 0.23 |
| TCGA-AA-3510-01 | Colorectal Adenocarcinoma | E942* | Nonsense_ | Diploid | 0.37 |
| TCGA-EI-6917-01 | Colorectal Adenocarcinoma | R204* | Nonsense_ | Diploid | 0.22 |
| TCGA-AA-3510-01 | Colorectal Adenocarcinoma | R204* | Nonsense_ | Diploid | 0.18 |
| TCGA-AA-3864-01 | Colorectal Adenocarcinoma | A83T | Missense_ | Diploid | 0.27 |
| TCGA-F5-6811-01 | Colorectal Adenocarcinoma | Q682* | Nonsense_ | Gain | 0.36 |
| TCGA-D5-6922-01 | Colorectal Adenocarcinoma | R247* | Nonsense_ | Diploid | 0.28 |
| TCGA-DM-A1HB-01 | Colorectal Adenocarcinoma | V940Sfs*5 | Frame_Shift_Del | Diploid | 0.21 |
| TCGA-G4-6304-01 | Colorectal Adenocarcinoma | V940Sfs*5 | Frame_Shift_Del | Diploid | 0.13 |
| TCGA-AG-A02N-01 | Colorectal Adenocarcinoma | Q748R | Missense_ | Diploid | 0.34 |
| TCGA-AA-3984-01 | Colorectal Adenocarcinoma | M979I | Missense_ | Diploid | 0.26 |
| TCGA-AA-3693-01 | Colorectal Adenocarcinoma | N310K | Missense_ | Diploid | 0.14 |
| TCGA-AA-A00N-01 | Colorectal Adenocarcinoma | R369I | Missense_ | Diploid | 0.26 |
| TCGA-AA-A010-01 | Colorectal Adenocarcinoma | P287L | Missense_ | Diploid | 0.31 |
| TCGA-AA-3692-01 | Colorectal Adenocarcinoma | R1050Q | Missense_ | ShallowDel | 0.31 |
| TCGA-AG-A002-01 | Colorectal Adenocarcinoma | P1046H | Missense_ | Diploid | 0.37 |
| TCGA-5M-AAT6-01 | Colorectal Adenocarcinoma | R840G | Missense_ | Diploid | 0.09 |
| TCGA-CA-6717-01 | Colorectal Adenocarcinoma | X913_splice | Splice_Site | Diploid | 0.12 |
| TCGA-NH-A50V-01 | Colorectal Adenocarcinoma | R136H | Missense_ | Diploid | 0.23 |
| TCGA-F5-6814-01 | Colorectal Adenocarcinoma | R187* | Nonsense_ | Diploid | 0.44 |
| TCGA-A6-2686-01 | Colorectal Adenocarcinoma | C411S | Missense_ | Diploid | 0.54 |
| TCGA-A6-3809-01 | Colorectal Adenocarcinoma | L386P | Missense_ | Diploid | 0.26 |
| TCGA-A6-6780-01 | Colorectal Adenocarcinoma | R786H | Missense_ | Diploid | 0.24 |
| TCGA-AA-3510-01 | Colorectal Adenocarcinoma | V430A | Missense_ | Diploid | 0.34 |
| TCGA-AA-3811-01 | Colorectal Adenocarcinoma | I212del | In_Frame_Del | Diploid | 0.31 |
| TCGA-AY-6197-01 | Colorectal Adenocarcinoma | Q787* | Nonsense_ | Diploid | 0.42 |
| TCGA-AY-6197-01 | Colorectal Adenocarcinoma | K305Nfs*16 | Frame_Shift_Del | Diploid | 0.31 |
| TCGA-AZ-4315-01 | Colorectal Adenocarcinoma | A129T | Missense_ | Diploid | 0.35 |
| TCGA-D5-6533-01 | Colorectal Adenocarcinoma | E86* | Nonsense_ | Gain | 0.41 |
| TCGA-DM-A28E-01 | Colorectal Adenocarcinoma | L645V | Missense_ | Diploid | 0.39 |
| TCGA-G4-6299-01 | Colorectal Adenocarcinoma | R302H | Missense_ | Diploid | 0.47 |
| TCGA-DY-A1DC-01 | Colorectal Adenocarcinoma | R204G | Missense_ | Diploid | 0.32 |
| TCGA-EI-6917-01 | Colorectal Adenocarcinoma | A1002V | Missense_ | Diploid | 0.11 |
| TCGA-EI-6917-01 | Colorectal Adenocarcinoma | R247Q | Missense_ | Diploid | 0.36 |
| TCGA-A4-7732-01 | Renal Non-Clear Cell Carcinoma | S179C | Missense_ | Diploid | 0.55 |
| TCGA-B9-A8YI-01 | Renal Non-Clear Cell Carcinoma | Y654F | Missense_ | Diploid | 0.33 |
| TCGA-IQ-A6SG-01 | Head and Neck Squamous Cell Carcinoma | Y1055C | Missense_ | Gain | 0.47 |
| TCGA-T3-A92N-01 | Head and Neck Squamous Cell Carcinoma | I605F | Missense_ | ShallowDel | 0.6 |
| TCGA-CV-7248-01 | Head and Neck Squamous Cell Carcinoma | A598S | Missense_ | Gain | 0.23 |
| TCGA-T2-A6X2-01 | Head and Neck Squamous Cell Carcinoma | E303K | Missense_ | Diploid | 0.31 |
| TCGA-D6-A4ZB-01 | Head and Neck Squamous Cell Carcinoma | S664* | Nonsense_ | Gain | 0.06 |
| TCGA-CQ-5331-01 | Head and Neck Squamous Cell Carcinoma | R1060L | Missense_ | Diploid | 0.16 |
| TCGA-P3-A6T5-01 | Head and Neck Squamous Cell Carcinoma | C171Y | Missense_ | ShallowDel | 0.19 |
| TCGA-H7-A6C4-01 | Head and Neck Squamous Cell Carcinoma | X581_splice | Splice_Region | ShallowDel | 0.11 |
| TCGA-IR-A3LK-01 | Cervical Squamous Cell Carcinoma | X887_splice | Splice_Site | Diploid | 0.43 |
| TCGA-EA-A3HS-01 | Cervical Squamous Cell Carcinoma | R141C | Missense_ | Diploid | 0.18 |
| TCGA-IR-A3LA-01 | Cervical Adenocarcinoma | Q546* | Nonsense_ | ShallowDel | 0.86 |
| TCGA-DS-A0VM-01 | Cervical Squamous Cell Carcinoma | E366K | Missense_ | Diploid | 0.26 |
| TCGA-2W-A8YY-01 | Cervical Squamous Cell Carcinoma | G314S | Missense_ | Diploid | 0.32 |
| TCGA-2W-A8YY-01 | Cervical Squamous Cell Carcinoma | R141H | Missense_ | Diploid | 0.32 |
| TCGA-2W-A8YY-01 | Cervical Squamous Cell Carcinoma | L297P | Missense_ | Diploid | 0.23 |
| TCGA-ZJ-AB0I-01 | Cervical Squamous Cell Carcinoma | R1007T | Missense_ | Diploid | 0.32 |
| TCGA-HC-A6HY-01 | Prostate Adenocarcinoma | USP28-TMPRSS2 | fusion | Diploid | NA |
| TCGA-EJ-7785-01 | Prostate Adenocarcinoma | MAPRE1-USP28 | fusion | Diploid | NA |
| TCGA-HC-7232-01 | Prostate Adenocarcinoma | SNX19-USP28 | fusion | DeepDel | NA |
| TCGA-GN-A26D-06 | Melanoma | USP28-DRD2 | fusion | Amp | NA |
